# Theoretical Study on the Alkylimino-Substituted Sulfonamides with Potential Biological Activity

**DOI:** 10.1021/acs.jpcb.3c01965

**Published:** 2023-07-21

**Authors:** Jakub Brzeski, Aleksandra Ciesielska, Mariusz Makowski

**Affiliations:** Faculty of Chemistry, University of Gdańsk, Wita Stwosza 63, 80-308 Gdańsk, Poland

## Abstract

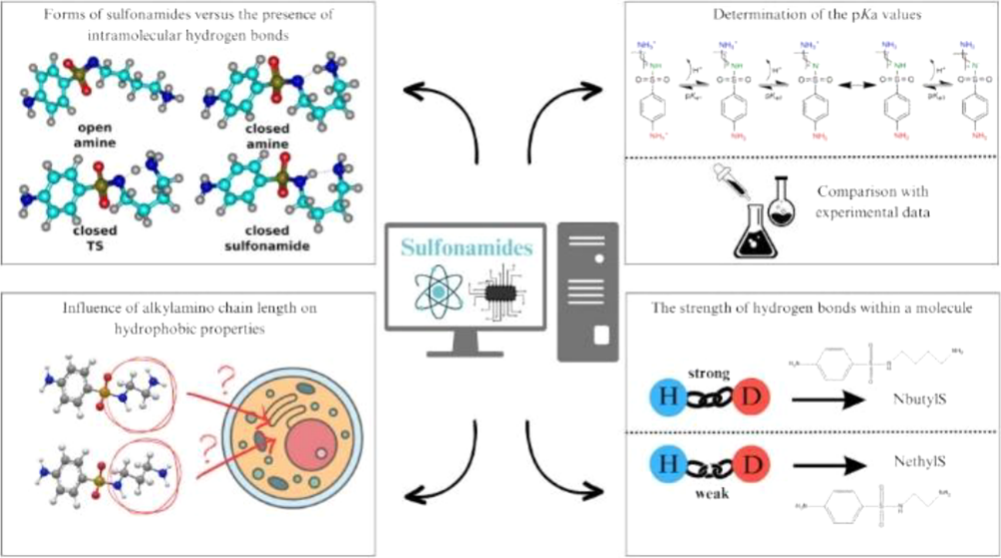

Antibiotics play a key role in the fight against bacterial diseases. However, bacteria quickly learn how to minimize the effects of antibiotics and strengthen their resistance. Thus, the fight against them becomes more and more difficult and there is a constant search for new bactericidal compounds. It is important in this type of search to determine the basic properties of compounds such as p*K*_a_, hydrogen bond formation, or hydrophobicity. Here, we present the results of our in silico study of five sulfonamide derivatives differing in alkylamine substituent length. Based on our results, we propose a model of three possible p*K*_a_ values for each of the studied compounds. Interestingly, the use of Muckerman’s approach for p*K*_a_ determination exhibits that theoretical and experimental results are in very good agreement. Intramolecular hydrogen bond formation affects p*K*_a_. The strength of the H-bond interaction increases from ethyl to butylamine and then decreases with the elongation of the alkylamine chain. The obtained partition coefficients (expressed here in the value of log *P*) increase with the number of carbon atoms in the alkylamine chain following Lipinski’s rule of five. The presented results provide important structural, physicochemical, and thermodynamic information that allows for the understanding of the influence of some sulfonamides and their possible activity.

## Introduction

Sulfa drugs comprise a wide range of pharmaceuticals used for the treatment of various diseases. Among others, those characterized by antibacterial (e.g., Sulfathiazole^1^), anticancer (e.g., E7070^2^), and antiviral (e.g., Amprenavir^3^) seem to attract the most attention. Although this group of pharmaceuticals exhibits a broad range of biological activity, it is mostly known for its antimicrobial properties. These are based on structural similarity to *para*-aminobenzoic acid (PABA) with which it competes for the binding to dihydropteroate synthetase (DHPS) enzyme and thus prevents the synthesis of bacterial dihydrofolic acid.^4^ This in turn prevents the replication of bacteria. One of the most important sulfa drugs is sulfamerazine, which, combined with trimethoprim, is used in the treatment of diseases like bronchitis, pneumonia, and urinary infections.^5^ Moreover, there are records claiming that it has chemotherapeutic activity as well.^6^ Another sulfa drug that is currently being used in the treatment of *dermatitis herpetiformis* (Duhring’s disease) is sulfapyridine.^7^ The versatility of medical effects exhibited by pharmaceuticals based on sulfanilamide derivatives is indicative of their therapeutic potential.

The process of designing a new drug is complicated and involves selecting the appropriate properties of the compound depending on its site of action and the route of administration. Particular attention should be paid to the structure, particle size, and stability of chemicals, biological agents, and metabolite products. Moreover, it is also very important hydrophilic–lipophilic nature of the compound, its solubility, acid–base properties, degree of ionization, thermal nature, activity, and cytotoxicity. In the aqueous environment, polar compounds are easily excreted by the kidneys but may have difficulty with the barrier of lipid membranes. However, lipophilic compounds may not penetrate the blood, and if they do get there, they are absorbed by fat cells. However, regardless of the mode of delivery used, water solubility will be required to ensure that the active molecules can achieve their desired goals, for example, solubility in the gastrointestinal tract, blood plasma, or lung fluid. Taken together, a prototype drug molecule that has a better chance of reaching the target will be soluble, moderately lipophilic, and have sufficient structural features to effectively interact with the target without unduly interfering with the many other functional molecules and macromolecules it encounters.^8^

According to the *Outpatient Antibiotic Prescriptions* report from 2020, the most often prescribed classes of antibiotics in the United States were (with the number of prescriptions given in parentheses) penicillins (43 mln), cephalosporins (30 mln), macrolides (29 mln), tetracyclines (23 mln), and β-Lactams with increased activity (21 mln).^9^ Less frequently prescribed antibiotic groups, including sulfonamides, were not listed in the aforementioned report. The former widespread use of sulfonamide drugs (which are in use since the 1930s) is currently limited, mostly due to the widespread resistance.^10^ The other factors affecting their current and somewhat scarce application, as compared to former commonness, are the side effects associated with their use^11^ and the availability of different groups of antibiotics. It is worth noting here that the same reason for the decline of the application of sulfonamide antibiotics affects every other antibiotic class nowadays. The overuse of discussed drugs and the paucity of new antibiotics have led to a global crisis.^12^

**Figure 1:**
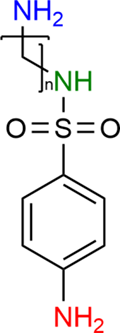
General formula of sulfonamides analyzed in this study, where n is the number of **–CH_2_–** groups within the structure; *n* = 2–6.

Because of the continuous enhancement of antibiotics resistance in general, new sulfonamides may be regarded as promising antimicrobial drugs. This is due to both inexpensive and relatively simple synthesis, as compared to other antibiotic groups. The synthesis of novel sulfa drugs ought to be followed by a scrupulous assessment of both physicochemical properties and biological activity. The particular stress should be laid on the side effects of the aforementioned species, with proper conclusions drawn from past research on sulfonamide pharmaceuticals. This paper is an attempt to lay a foundation for described experimental endeavors because it describes the physicochemical properties of 5 sulfonamides (4-amino-N-(2-aminoethyl)benzenesulfonamide–NethylS, 4-amino-N-(2-aminopropyl)benzenesulfonamide–NpropylS, 4-amino-N-(2-aminobutyl)benzenesulfonamide–NbutylS, 4-amino-N-(2-aminopentyl)benzenesulfonamide–NpentylS, and 4-amino-N-(2-aminohexyl)benzenesulfonamide–NhexylS) with the predominant use of quantum chemical methods. All sulfonamides regarded in this paper differ in the alkylamine substituent length (see Figure 1 below). The alkylamine moiety itself is present in various drugs exhibiting a wide range of activities, such as an antituberculosis agent in Ethambutol^13^ or antiasthmatic in Aminophylline.^14^ The parameters reported herein are crucial for a correct assessment of the activity of analyzed sulfonamides. Our analysis provides the structural and energy differences between studied molecules and their isomers, acid–base equilibria, intramolecular hydrogen bonds, and hydrophobicity.

The geometrical structure of sulfa drugs determines their biological reactivity as it influences the binding to the receptor, e.g., dihydropteroate synthetase. The differences in electronic energies (or Gibbs free energies) between isomers regulate the population of each isomer at the given conditions. Since isomers of various pharmaceuticals have been shown to exhibit disparate biological activity,^15^ the said analysis appears crucial. The protonation state, on the other hand, except for affecting activity, influences the permeation of various membranes within the human body. Values of p*K*_a_ allow for the prediction of the protonation form of the pharmaceutical at each step of the metabolic pathway, which is crucial in the design of therapy. Another important factor affecting the reactivity is the partition coefficient (expressed here in the value of log *P*), which allows for the assessment of the distribution of a given pharmaceutical within the body. The investigation of the intramolecular hydrogen bond, on the other hand, gives an insight into both the acidity and hydrophobicity of the studied compounds.

## Methods

**Figure 2:**
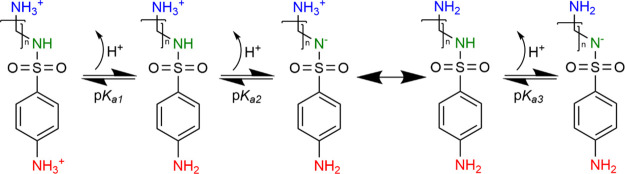
Acid–base equilibria for alkylamine-substituted sulfonamides studied in this paper, where *n* = 2–6.

The dissociation constant of the acid provides information about the protolytic form; therefore, we proposed the following dissociation equilibria (Figure 2), the validity of which is described in the next part of the publication.

The equilibrium structures of all compounds were obtained by applying meta-GGA approximation-based hybrid M06 functional^16^ with split–valence triple-ζ Pople-type basis set–6-311++G(2d,2p).^17,18^ The Berny (with GEDIIS) algorithm was used during the minimization.

The influence of solvents (water with ε = 78.3553 and n-octanol with ε = 9.8629) was approximated by the application of the Self-Consistent Reaction Field (SCRF) method^19^ and the SMD model.^20^ The harmonic vibrational frequencies characterizing the stationary points were evaluated analytically at the very same level of theory to assure that all of the obtained structures correspond to true minima or first-order saddle points (TS) on the potential energy surface. The intrinsic reaction coordinate (IRC) procedure^21,22^ was applied to confirm the minima for each TS.

**Figure 3:**
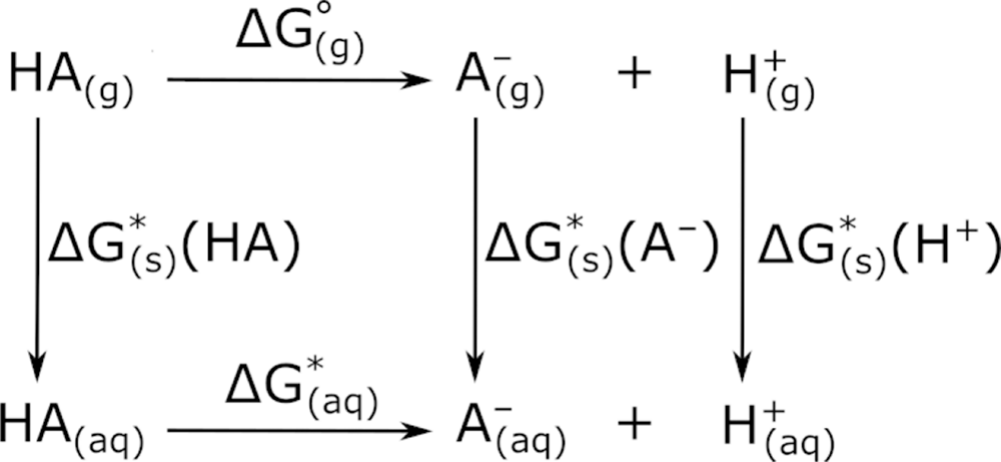
Thermodynamic cycle used^23^ for the calculation of p*K*_a,calc_ according to the direct method. The ‘°’ superscript denotes the condition of 1 atm, whereas the ‘*’ represents 1 M.

The values of p*K*_a,calc_ corresponding to various protonation states of each sulfonamide were calculated with the direct method,^23,24^ which employs the use of a thermodynamic cycle presented in Figure 3 below:

The change of Gibbs free energy of deprotonation (Δ*G*_(aq)_) was calculated according to eq 1:
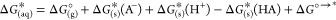
1where Δ*G*_(s)_^*^(A^–^), Δ*G*_(s)_^*^(H^+^), and Δ*G*_(s)_^*^(HA) are the standard-state solvation free energies of A^–^, H^+^, and HA, respectively, and Δ*G*_(g)_^°^ represents the Gibbs free energy of the gas-phase deprotonation of HA:
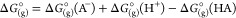
2The Δ*G*^°→*^ term guarantees the same standard conditions to both phases, as it converts 1 atm of an ideal gas standard state to an 1 M aqueous standard state. At 298 K, the Δ*G*^°→*^ assumes the value of 1.89 kcal/mol.^25^ Values of Δ*G*_(aq)_^*^ were used to calculate p*K*_a,calc_ according to the equation:
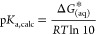
3

Since p*K*_a,calc_ values calculated this way tend to deviate from experimental results to some extent, we have decided to also use the approach proposed by Muckerman et al.^26^ In this method, a certain number of experimental results is necessary to introduce the p*K*_a,calc_ “lift factor” that was obtained here separately for each equilibrium and later on used to correct the computationally determined values:
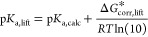
4where Δ*G*_corr, lift_^*^ is a parameter correcting the calculated values of Gibbs free energies based on the results obtained experimentally as described by Muckerman et al.^26^

Muckerman et al. observed^26^ that the p*K*_a_ values obtained based on the thermodynamic cycle were poorly reproduced. They claimed that most of the error arose from inaccurate differential solvation free energies of the acid and conjugated base. To eliminate that they proposed in their approach a correction based on the realization that the gas-phase acidities had only a small systematic error relative to the dominant systematic error in the differential solvation. They illustrated the insensitivity of their approach to the functional. Their method could be applied to the comparison of results for sets of neutral acids and protonated amine cationic acids in both aqueous (water) and nonaqueous (acetonitrile) solvents, We have also proven that it worked for pyridine and its N-oxide derivatives in water and acetonitrile.^24^

The hydrophilicity of the sulfa drugs was assessed by the calculation of log *P* values, according to the following equation:^27^
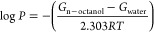
5In eq 5, Δ*G*_n–octanol_ and Δ*G*_water_ correspond to the Gibbs free energies of an electrically neutral solute in n-octanol and water. As can be seen from eq 3, the lower the value of log *P*, the more hydrophilic the corresponding solute.

The energies of intramolecular hydrogen bonds (*E*) were assessed using the method proposed by Espinosa et al.,^28^ i.e., according to the following equation:
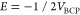
6where *V*_BCP_ stands for a potential energy density of a Bond Critical Point (BCP) corresponding to the interaction of interest.

The Fuzzy Bond Order (FBO)^29^ analysis was performed to assess the degree of covalence of studied hydrogen bonds.^30^ In this approach, the bond order is calculated according to the following formula, eq 7:
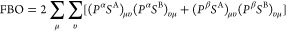
7

In the formula above *P* is the density matrix, *S* stands for the overlap matrix of basis functions in fuzzy atomic spaces, A and B indices represent atoms forming a given bond, and α and β stand for a spin, where μ and ν are basis orbitals indices. The concept of “fuzzy” atoms has appeared for the first time in the scientific discourse with the study by Hirshfeld.^31^ The mentioned study describes a division of 3D molecular space into atomic regions corresponding to separate atoms having no definitive boundaries, yet being continuously connected. In Mayer’s FBO,^32^ the fuzzy atomic regions arise from the introduction of a weight function defining the division of a 3D space into “fuzzy” regions.

The biggest value of the error of atomic overlap matrix (AOM) in FBO calculations was found to be equal to 0.00165. Both the QTAIM and FBO analyses were performed using the Multiwfn^33^ software.

The final cartesian coordinates of all studied compounds can be found in the Supporting Information (Table S1). All quantum chemical calculations were carried out using the GAUSSIAN09 (Rev.C.01) package.^34^

### Experimental Details of Studies of the NbutylS Compound

Two sulfonamide derivatives NethylS and NpropylS were physicochemically characterized previously and the results of those studies were published elsewhere.^35^ To extend the scope of the experiment, a third N-butylS compound was synthesized, whose characterization has not been published so far. The NbutylS derivative has the longest alkylamino chain and is more basic. Its chain is more flexible than ethyl and propyl derivatives and finally is also more hydrophobic than the remaining two derivatives.

The synthesis of the NbutylS and determination of its p*K*_a_ values were carried out analogously to the procedures described elsewhere.^35^ Elemental analysis, nuclear magnetic resonance (NMR), mass spectrometry (MS), and Fourier transform infrared spectroscopy (FTIR) were used to confirm the structure of the compound, the results of which are presented in the Supporting Information (Figures S2–S4).

The study of the acid–base properties of the compound was performed using pH-spectrometric titration. Acid dissociation constant (p*K*_a_) values were calculated with the form of the Henderson–Hasselbach eq 8 implemented into Origin Lab software:
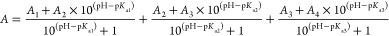
8

**Figure 4:**
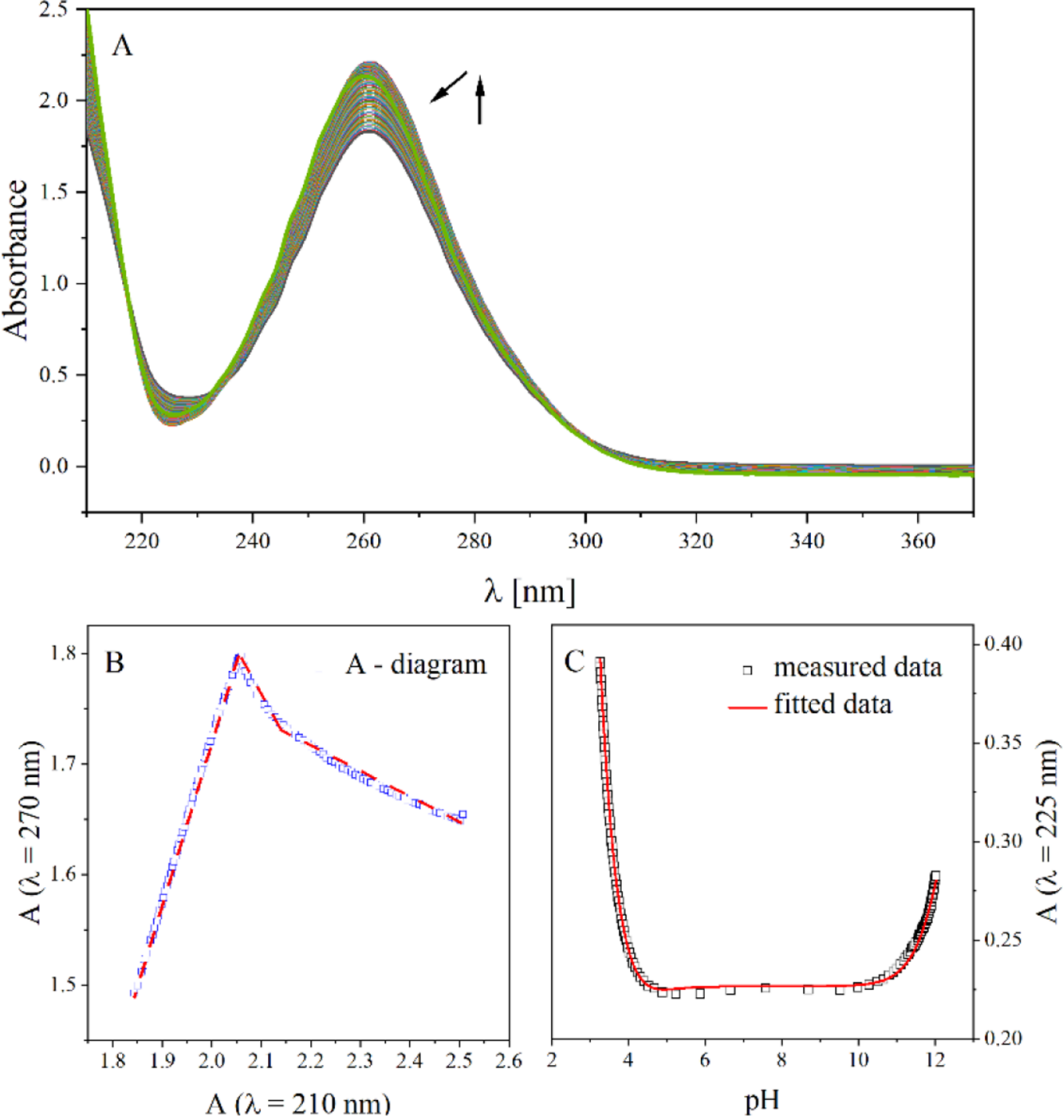
(A) Spectrophotometric acid–base titration curves of NbutylS. The arrows indicate the direction of changes in the intensity of the absorption band. (B) A-diagram presents absorbance change at 270 nm from absorbance change at 210 nm. (C) Absorbance (A) of a compound as a function of the pH at λ = 255 nm.

In the graph of the titration process (Figure 4A), spectral changes such as intensity change and hypsochromic shift are observed. From the collected experimental data, an A-diagram (Figure 4B) is plotted which shows the relationship between the absorbance at 270 nm and the absorbance at 210 nm. Based on this, the number of equilibria in the solution for the analyzed compound was determined. The presence of three equilibria in the solution was proved, as evidenced by the presence of three segments in the A-diagram (Figure 4B) and the same number of absorbance inflections (A) as a function of the pH curves (Figure 4C). The calculated p*K*_a_ values of the NbutylS compound are summarized in table (Table 1).

**Table 1:** Wavelengths Maxima (λ_max_) and Acid Dissociation Constant (p*K*_a_) Values (with Standard Deviation) Determined by the pH-Spectrophotometric Titration Method in Water

λ_max_ [nm]	λ_max_ [nm] (with acid)^a^	λ_max_ [nm] (with alkali)^a^	p*K*_a1_	p*K*_a2_	p*K*_a3_
261	261	260	1.76 ± 0.07	4.51 ± 0.14	12.28 ± 0.04

a The values of wavelength maxima in the acidic environment, i.e., the pH before starting the titration process, and in the alkaline environment, i.e., the pH at which the titration was completed, are given.

## Results and Discussion

### Acid–Base Equilibria

**Figure 5:**
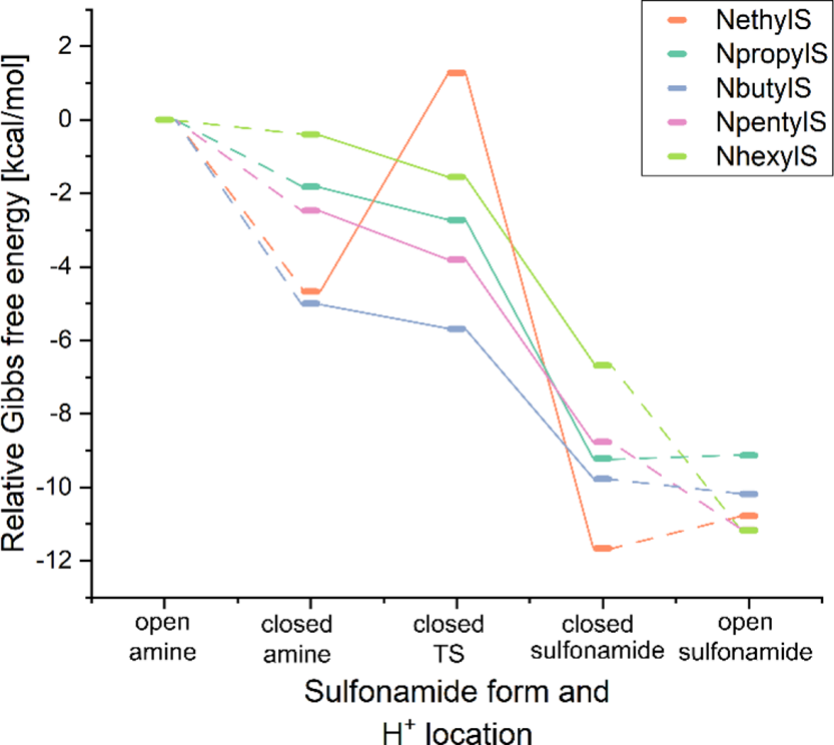
Relative Gibbs free energies of the electrically neutral forms of the studied sulfonamides.

As mentioned before, the sulfonamides with alkylamine substituents that have been tested here differ structurally in the length of the (−CH_2_−)_*n*_ chain. Namely, there are compounds with *n* = 2–6 methylene groups separating the sulfonamide moiety from the amino group. As experimental results suggested, the deprotonation of particular functional groups of studied sulfonamides should occur in the following order: aromatic amine, sulfonamide, and aliphatic amine at the end.^35^ Theoretical calculations shed new insights into the said equilibria. Interestingly, it was found here that for the mentioned order of deprotonation, the calculations of the p*K*_a_ values for the last two equilibria have led to unexpected results, as the values of p*K*_a3_ were calculated to be lower than the corresponding values of p*K*_a2_. A closer examination of the ongoing process has shown that the zwitterion formed after the second deprotonation process (deprotonation of sulfonamide group) may undergo a geometrical rearrangement. Namely, the formation of an intramolecular hydrogen bond between aliphatic amine and deprotonated sulfonamide group and thus closing of the otherwise mobile alkylamine chain may occur (see Figure 5). The formed hydrogen bond may be regarded as a clasp bringing about a second cycle in the molecule.

As is evident from Figure 5, the formation of an intramolecular hydrogen bond, and thus the ‘closing” of sulfonamide is favored thermodynamically regardless of the system considered. In the next step, the acidic proton may be transferred from the alkylamine to the sulfonamide group. This process is expected to be thermodynamically barrierless for each of the studied sulfonamides but NethylS. For NethylS, the barrier was calculated to be equal to 5.9 kcal/mol, which suggests that it may easily occur in standard conditions. The reasons for the existence of the proton transfer barrier in NethylS are discussed in the succeeding part of the manuscript. The analyzed proton transfer leads to the regeneration of the protonated sulfonamide group. The considered structures, in which the N atom of the sulfonamide group acts as a hydrogen bond donor whereas that of the alkylamine groups is an acceptor, are described by lower values of Gibbs free energy than any other structure hitherto discussed. It is important to realize, however, that the situation may be different in a real solution, especially in the case of polar solvents, where the zwitterionic form (see Figure S1) may be favored. To get a complete picture of the occurring processes, the straightening of the thus-obtained alkylamine chain (and thus breaking of intramolecular hydrogen bonds) was also investigated. As is evident from Figure 5, the unfolding of the alkylamine chains is favored thermodynamically for all but two of the studied compounds, i.e., NethylS and NpropylS. For the remaining cases, strain energy coming from the bending of the alkylamine chain to form a hydrogen bond (and thus close the sulfonamide) is higher than that of the formed hydrogen bond. Nonetheless, the rather insignificant differences in Gibbs free energies for closed and opened forms of sulfonamides with H^+^ on the sulfonamide group indicate that in standard conditions, both forms are likely to be present. As can be seen in Figure 5, undoubtedly, the most favorable form of the neutral sulfonamides studied here is one with acidic H on the sulfonamide group, regardless of whether it is closed by the hydrogen bond or not. The exemplary structures of various forms of neutral sulfonamides are presented in Figure S1.

Bearing all of the above in mind, we propose the following deprotonation order: aromatic amine, sulfonamide deprotonation followed by an acidic H transfer from alkylamine to the sulfonamide group, and second deprotonation of sulfonamide (see Figure 2).

The values of p*K*_a_ corresponding to successive deprotonation steps are shown in Table 2. As can be seen from the said table, the values of p*K*_a_ calculated from the thermodynamic cycle (Figure 3) differ significantly from those determined experimentally. The differences are most evident for the first and last steps of deprotonation. The disparities presumably arise from the insufficient inclusion of solvent effects in the SCRF calculations. The application of the method proposed by Muckerman allows for a significant improvement in the results.^26^

**Table 2:** p*K*_a_ Values Calculated for the Studied Compounds^a^

system	p*K*_a1,calc_	p*K*_a1,lift_	p*K*_a1,exp_	p*K*_a2,calc_	p*K*_a2,lift_	p*K*_a2,exp_	p*K*_a3,calc_	p*K*_a3,lift_	p*K*_a3,exp_
NethylS	–4.77	2.58	2.62 ± 0.19^b^	8.15	6.14	6.45 ± 0.21^b^	18.29	12.07	9.67 ± 0.08^b^
NpropylS	–4.35	2.27	2.11 ± 0.10^b^	8.78	6.68	6.35 ± 0.06^b^	14.92	9.41	10.86 ± 0.19^b^
NbutylS	–4.42	1.70	1.76 ± 0.07^c^	8.32	4.49	4.51 ± 0.14^c^	17.16	11.33	12.28 ± 0.04^c^
NpentylS	–4.39	1.75	1.76^d^	8.36	6.88	6.79^d^	17.20	10.45	e
NhexylS	–5.90	1.14	1.19^d^	11.72	8.24	8.04^d^	16.91	10.33	e

a Values marked by *calc* in subscript come from the thermodynamic cycle-based calculations, whereas those marked with *lift* were obtained with the use of Muckerman’s approach.^26^ Experimental results are designated with *exp*. and their standard deviations are also provided.

b Values from ref (32).

c Values obtained in this work.

d Values extrapolated from eqs 9 and 10.

e Values are indeterminable due to the low correlation between p*K*_a3,exp,_ and p*K*_a3,lift_.

An analysis of the p*K*_a1_ allows to conclude that elongation of the alkylamine chain leads to the increase in the acidity of the aromatic amine group. The effect, however, is rather limited, which is of no surprise bearing in mind the distance separating the two functional groups in question. For the second step of deprotonation, i.e., p*K*_a2_, the results of theoretical calculations seem to show a different trend than experimental ones. Namely, according to calculations, values of p*K*_a2_ should increase with the length of the alkylamine chain. Whereas experimental results (for which the available data are limited to three compounds) exhibit the opposite tendency. The p*K*_a2_ values corresponding to NbutylS are an exception in both approaches, as they are determined to be equal to ∼4.5 as opposed to ∼6.5 observed for remaining sulfonamides. This is likely due to the fact that for singly deprotonated (via aromatic amino group) NbutylS, there is a stabilizing intramolecular hydrogen bond between the −NH_3_^+^ alkylamine group and the O atom of the sulfonamide moiety. A shift of the electron density from the sulfonamide group to the H atom of the alkylamine group leads to the increase of the acidity of the first, rendering the p*K*_a2_ of NbutylS almost two units lower than that of remaining sulfonamides. Additionally, the product of the said deprotonation is also stabilized by the strongest of hydrogen bonds studied here. For the last step of deprotonation, i.e., p*K*_a3_, calculations, and experiment again show the opposite trend. Namely, calculations suggest that the values of p*K*_a3_ should be expected to decrease with the increasing number of methylene groups in the alkyl chain, whereas the experiment shows an increase from 9.67 to 12.28 while going from NethylS to NbutylS. As mentioned earlier, the discrepancies between theoretical and experimental results arise from insufficient inclusion of solvent effects. The other important factor that stands in the way of obtaining accurate values of p*K*_a_ with the use of theoretical methods is the omission of the presence and thus influence of various forms of compounds in a real solution. By definition, the p*K*_a_ is calculated using only two equilibrium structures, one of the acid and second of the conjugate base. However, it is worth adding that in a real solution, compounds appearing as various conformers (or isomers) are present. Therefore, a hypothetical theoretical model that accurately predicts the values of p*K*_a_ would necessarily need to account for all types of systems present in the solution. The obtained values of p*K*_a,lift,_ and available data on p*K*_a,exp_ were used to assess the p*K*_a1,exp_ (eq 9) and p*K*_a2,exp_ (eq 10) values for NpentylS and NhexylS. The values of p*K*_a3,exp_ could not be determined in the same manner, as the correlation between p*K*_a3,lift,_ and p*K*_a3,exp_ is rather limited.
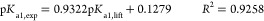
9
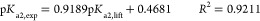
10

Our calculations suggest that the p*K*_a1,exp_ of NpentylS should be close to that of NbutylS, whereas that of NhexylS might be as low as 1.19. As for the p*K*_a2,exp_ values, the values for NpentylS and NhexylS were calculated to be equal to 6.79 and 8.04 respectively, indicating a decreasing acidic strength of the corresponding sulfonamide group.

Following Muckerman’s claim, we might conclude that the methodology allows to generally predicts p*K*_a_ values for all the cases investigated within 1 p*K*_a_ unit (this is also observed in our case for p*K*_a1_ and p*K*_a2_) as the differential solvation error is larger than the systematic error in the gas-phase acidity calculations. Oppositely, p*K*_a3_ differs by more than 1 p*K*_a_ unit from those experimental, suggesting that the systematic error in the gas-phase acidity calculations dominates.

### Intramolecular Hydrogen Bonds

As mentioned in the previous paragraph, alkylamine and sulfonamide groups can form an intramolecular hydrogen bond leading to a closure of the otherwise mobile alkylamine chain. Due to the fact, that the nonzwitterionic form of the electrically neutral sulfonamides is lower in energy than its zwitterionic form (Figure S1), the first one of each studied sulfonamide was subjected to a detailed examination. It is apparent from Table 3 that the strongest hydrogen bond is formed for NbutylS, whereas the weakest interaction was calculated for NethylS. For NethylS, the shortest D–H (1.022 Å), the longest H···A (2.392 Å), and the smallest values of the <(DHA) dihedral angle (106.4 deg) are observed. Because of the said geometrical limitations, the formed hydrogen bond is so weak that it cannot be detected via topology of electron density analysis, as no bond critical points are associated with the interaction. The situation is analogous in the case of FBO analysis.

**Table 3:** H-Bond Distances (in Å), Angles (in deg), Energies (in kcal/mol), and FBO in Closed Electrically Neutral Form of Sulfonamides

system	d(D–H)	d(H···A)	d(D···A)	<(DHA)	E	FBO^a^
NethylS	1.022	2.392	2.855	106.4	–b	–b
NpropylS	1.028	2.066	2.877	134.0	5.08	0.080
NbutylS	1.040	1.846	2.842	159.3	9.16	0.124
NpentylS	1.036	1.948	2.966	167.0	6.55	0.101
NhexylS	1.036	1.964	2.999	177.5	6.16	0.102

a Dimensionless.

b Values could not be determined.

NbutylS is on the other side of the energetic spectrum, as the energy calculated for H-bond is equal to 9.16 kcal/mol. The H-bond of NbutylS is characterized by the lowest value of H···A (1.846 Å) and the highest value of the D–H bond length (1.040 Å) of all studied compounds. Additionally, the value of FBO is equal to 0.124, which is ca 22% higher than the one corresponding to the H-bond with the second highest FBO calculated for NethylS. In the case of the discussed compound, the formation of an intramolecular H-bond leads to the creation of a 7-membered ring (Figure S1), made out of NH···N and four C atoms. Both values of E and FBO indicate that for NbutylS, the energy of the H-bond surpasses the one arising from the alkylamine chain strain by the greatest margin.

Naturally, the H-bonds corresponding to remaining sulfonamides are described by values in-between these mentioned earlier. Namely, the hydrogen bond lengths vary from 1.964 to 2.066 Å, and the energies change from 5.08 to 6.55 kcal/mol, whereas FBO takes values from 0.080 to 0.102. The general trend that can be noticed is that the strength of the H-bond interaction increases from *n* = 2 to *n* = 4 and then decreases with the elongation of the alkylamine chain.

### Hydrophobicity

As mentioned earlier, hydrophobicity is an important parameter describing biologically active compounds. As shown in Table 4, values of log *P* theoretically obtained were collected. As anticipated, the hydrophobicity of the studied compounds increases with the number of carbon atoms in the alkylamine chain. Furthermore, the relaxation of the alkylamine chain (preceded by the breaking of the H-bond) leads to a decrease in hydrophobicity. The said decrease seems to be to some extent proportional to the length of the alkylamine chain, as for NethylS, it is only 0.47 and for NhexylS, it is as big as 3.02 units.

**Table 4:** Calculated Values of log *P* for Closed and Open (in Parentheses) Forms of Nonzwitterionic Sulfonamides

system	log *P*
NethylS	–3.76 (−4.23)
NpropylS	–3.15 (−3.56)
NbutylS	–0.94 (−2.65)
NpentylS	0.10 (−3.12)
NhexylS	1.53 (−1.49)

According to Lipinski’s rule of five,^36^ an orally active compound has a log *P* value not exceeding 5. As such, all compounds studied here may be regarded as orally active. The situation is somewhat different when considering the Ghose filter,^37^ according to which druglike compounds should be characterized by log *P* values in the range of −0.4 to 5.6. In light of that, only NpentylS and NhexylS in a closed form can be regarded as druglike.

## Conclusions

This study has investigated the set of alkylamine-substituted sulfonamides with potential biological activity with the use of computational methods. Taken together, we defined the following theses:

(i) The studied sulfonamides exist in forms differing in the presence (or absence) of intramolecular hydrogen bonds;
(ii) Electrically neutral sulfonamides may exist in zwitterionic and nonzwitterionic forms, whereby the latter is thermodynamically favored.
(iii) Proton transfer that is associated with zwitterionic to nonzwitterionic form transition is expected to be a barrierless process;
(iv) The calculated values of p*K*_a_ significantly differ from those obtained experimentally. It is the application of Muckerman’s approach that renders the theoretical findings regarding acid–base properties meaningful;
(v) For electrically neutral forms of studied sulfonamides, the strongest H-bond is present in NbutylS, whereas that of NethylS is on the other side of the spectrum;
(vi) The hydrophobicity of studied systems increases along with the length of the alkylamine chain. The formation of intramolecular H-bonds also leads to an increase in hydrophobicity.

Our studies provide a solid basis and provide a lot of information that can be used in the design of new drugs. They explain the behavior of molecules in the aquatic environment, which allows conclusions to be drawn about their behavior in the cell. The presence of intramolecular hydrogen bonds in the structure of sulfonamide derivatives may cause differences in the mechanism of interaction of the compound with biomolecules, compared to molecules in which this bond does not occur. The determined p*K*_a_ values of the compounds allow one to determine in which protolytic form the sulfonamide will occur in the cell, at physiological pH. The calculated bond strengths within the analyzed molecules may also affect the mechanism of interaction of the compound with biomolecules. In addition, they may affect the complexing properties of the discussed sulfonamides with biologically significant metal ions, the combinations of which may also find potential use in pharmaceuticals. The specific hydrophobicity of the compounds suggests whether they are capable to penetrate biological membranes and also whether they will be excreted through the kidneys. This is a very important aspect considered when designing new pharmaceuticals because the journey of a drug molecule from the site of administration to the site of action is complex and involves many changes in the environment. In conclusion, the conducted research provides information useful for understanding the behavior of molecules and is the basis for further computational, experimental, and biological studies.

## Supporting Information Available

Cartesian coordinates of the calculated equilibrium structures of all studied compounds in water (SMD) together with the corresponding values of electronic and Gibbs free energies; equilibrium structures of various forms of zwitterions sulfonamides; mass spectrum for NbutylS; FT-IR spectra for NbutylS; and ^1^H NMR spectra for NbutylS (PDF)
