# Correction to “Theoretical Study on the Alkylamino-Substituted Sulfonamides with Potential Biological Activity”

**DOI:** 10.1021/acs.jpcb.3c05618

**Published:** 2023-09-07

**Authors:** Jakub Brzeski, Aleksandra Ciesielska, Mariusz Makowski

In our previous paper, we found that there was a typo in the title of our paper. This typo was also in the Supporting Information file. “Alkylimino” should be “Alkylamino”. The corrected title is as follows: *Theoretical Study on the Alkylamino-Substituted Sulfonamides with Potential Biological Activity*.

The change is reflected in the title of this Correction and in the attached Supporting Information file. It does not affect our results, discussion, and conclusions.

## Supporting Information Available

Cartesian coordinates of the calculated equilibrium structures of all studied compounds in water (SMD) together with the corresponding values of electronic and Gibbs free energies; equilibrium structures of various forms of zwitterions sulfonamides; mass spectrum for NbutylS; FT-IR spectra for NbutylS; and ^1^H NMR spectra for NbutylS (PDF)

