# Supplementary material for: Theoretical Study on the Alkylimino-Substituted Sulfonamides with Potential Biological Activity
Source: J Phys Chem B. 2023 Jul 21;127(30):6620–7. doi: 10.1021/acs.jpcb.3c01965 (PMC10405214; doi:10.1021/acs.jpcb.3c01965)
Supplement: Supplementary file 1 — jp3c01965_si_001.pdf [file jp3c01965_si_001.pdf]

SUPPLEMENTARY INFORMATION

A THEORETICAL STUDY ON THE ALKYLIMINO SUBSTITUTED SULFONAMIDES WITH POTENTIAL BIOLOGICAL ACTIVITY

Jakub Brzeski[a], Aleksandra Ciesielska, Mariusz Makowski[a,*]

[a]*Faculty of Chemistry, University of Gdańsk, Wita Stwosza 63, 80-308 Gdańsk, Poland*

ORCID:
0000-0003-4865-0152 (J. B.)
0000-0002-7342-722X (M. M.)
0000-0002-0268-4210 (A. C.)

[*]corresponding authors: mariusz.makowski@ug.edu.pl

Keywords: sulfonamides, pK_a, hydrophobicity, DFT

Table S1. Cartesian coordinates (in Å) of the calculated equilibrium structures of all studied compounds in water (SMD) together with corresponding values of electronic energy (E in Hartree) and Gibbs free energy (G in Hartree).

Species	Coordinates			
$NethylSH_2^+$<br>E=-1026.2432708<br>G=-1026.038302<br>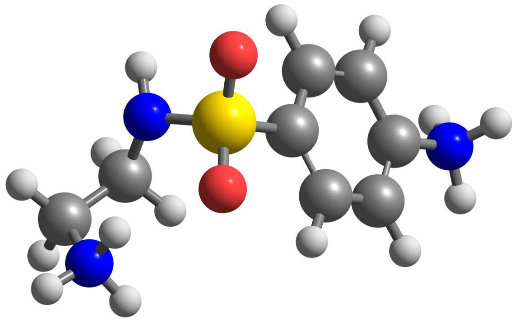	S	0.946628	1.062133	-0.335920
	O	0.907919	2.498335	-0.220858
	O	1.553204	0.440609	-1.491339
	N	-4.615529	-0.985279	0.173385
	C	-0.709234	0.446301	-0.206609
	C	-1.128929	-0.580223	-1.033419
	C	-1.543026	1.003720	0.753233
	C	-3.244356	-0.494991	0.044983
	C	-2.422556	-1.057998	-0.905593
	C	-2.831513	0.525780	0.880012
	H	-0.460895	-1.001792	-1.772125
	H	-1.194804	1.803381	1.395586
	H	-2.784120	-1.861245	-1.535916
	H	-3.508609	0.938455	1.617534
	H	-4.735000	-1.891366	-0.286914
	H	-5.283694	-0.333202	-0.249063
	N	1.771893	0.526372	0.974743
	C	1.900675	-0.919554	1.191864
	H	1.171789	-1.476626	0.593984
	H	1.665659	-1.123515	2.235310
	C	3.293038	-1.419008	0.919341
	H	4.038791	-0.802184	1.416744
	H	3.396972	-2.446411	1.261650
	N	3.604037	-1.408622	-0.538201
	H	4.576454	-1.669008	-0.705526
	H	3.436705	-0.485985	-0.951833
	H	-4.880033	-1.094321	1.156820
	H	1.513162	1.056476	1.801511
	H	3.005205	-2.069804	-1.037823
$NethylSH^+$<br>E=-1025.8087094<br>G=-1025.617942<br>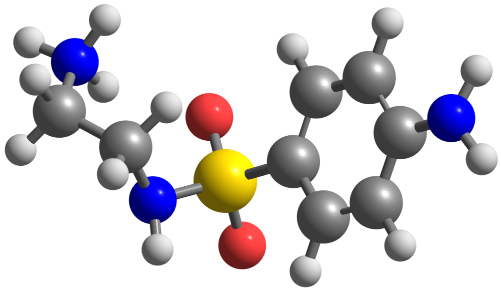	S	-0.897690	1.069248	0.352950
	O	-0.871668	2.511326	0.259319
	O	-1.528686	0.439180	1.496522
	N	4.558222	-1.068750	-0.235678
	C	0.728662	0.445833	0.220378
	C	1.150730	-0.613688	1.015530
	C	1.587071	1.004385	-0.724944
	C	3.306872	-0.553377	-0.065456
	C	2.429432	-1.104966	0.877039
	C	2.861593	0.511466	-0.865067
	H	0.479946	-1.044809	1.747762
	H	1.256781	1.828635	-1.347211
	H	2.770038	-1.924614	1.499338
	H	3.538636	0.943507	-1.593163
	H	4.929876	-1.637016	0.509869
	H	5.234718	-0.494318	-0.714602

	N	-1.755962	0.576039	-0.970545
	C	-1.847651	-0.862858	-1.239544
	H	-1.077714	-1.420616	-0.694346
	H	-1.650217	-1.020708	-2.298795
	C	-3.211763	-1.419010	-0.935048
	H	-3.995125	-0.806028	-1.376206
	H	-3.299345	-2.434163	-1.316407
	N	-3.463902	-1.477501	0.532783
	H	-4.426990	-1.750764	0.729659
	H	-3.277920	-0.572434	0.979440
	H	-1.473199	1.122880	-1.778388
	H	-2.841790	-2.156795	0.975872
NethylS-open/amine<br>E=-1025.3270496<br>G=-1025.150921<br>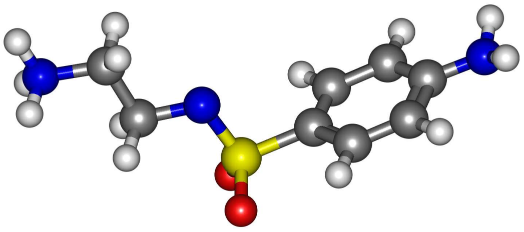	16	-0.640040	0.922593	0.227288
	8	-0.778096	2.059406	-0.710024
	8	-0.804288	1.387269	1.625370
	7	4.938836	-1.053515	-0.205964
	6	1.017313	0.328758	0.076164
	6	1.797880	0.670903	-1.017340
	6	1.541464	-0.494965	1.065554
	6	3.630714	-0.627111	-0.133480
	6	3.092321	0.198891	-1.123553
	6	2.831907	-0.969099	0.964084
	1	1.391929	1.311397	-1.790709
	1	0.935392	-0.766643	1.922389
	1	3.703618	0.467175	-1.978606
	1	3.242273	-1.610890	1.736379
	1	5.347677	-1.052349	-1.129126
	1	5.157255	-1.883660	0.325591
	7	-1.489702	-0.333887	-0.134099
	6	-2.916563	-0.115591	0.001187
	1	-3.285077	0.728622	-0.603370
	1	-3.221031	0.090269	1.039661
	6	-3.619555	-1.364812	-0.457546
	1	-3.419568	-1.574903	-1.506881
	1	-3.328305	-2.229063	0.137319
	7	-5.093822	-1.200117	-0.309712
	1	-5.595934	-2.035783	-0.610190
	1	-5.427687	-0.412420	-0.868665
	1	-5.340901	-1.015848	0.664836
NethylS-closed/amine<br>E=-1025.3366828<br>G=-1025.158358	S	-1.074934	-1.080640	-0.280137
	O	-1.011453	-2.542362	-0.382948
	O	-1.624775	-0.456637	-1.515420
	N	4.513182	0.913038	0.134980
	C	0.598893	-0.486109	-0.206937
	C	1.487635	-1.093283	0.674474
	C	1.013619	0.613862	-0.944038
	C	3.209053	0.477548	0.049636
	C	2.777714	-0.624833	0.797733
	C	2.304635	1.092425	-0.818963
	H	1.167460	-1.943409	1.266739

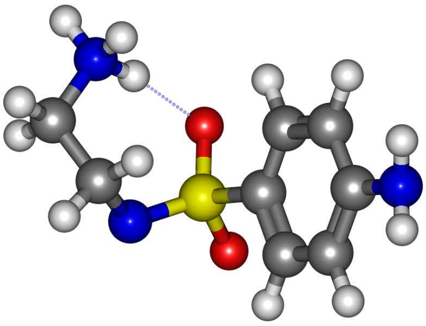	H	0.323335	1.099534	-1.623435
	H	3.472459	-1.105830	1.477820
	H	2.627259	1.951908	-1.396742
	H	5.003320	0.661708	0.980794
	H	4.674795	1.867404	-0.151138
	N	-1.812916	-0.704686	1.034158
	C	-1.827361	0.691767	1.429159
	H	-0.887945	1.219907	1.199024
	H	-1.918542	0.725232	2.517956
	C	-2.981825	1.507171	0.893409
	H	-3.915539	0.950008	0.957045
	H	-3.089541	2.439643	1.446007
	N	-2.776812	1.864662	-0.539887
	H	-3.606838	2.295686	-0.944530
	H	-2.518534	1.015091	-1.081394
	H	-2.001277	2.522961	-0.630621
NethylS-closed/TS<br>E=-1025.3217777<br>G=-1025.148891<br>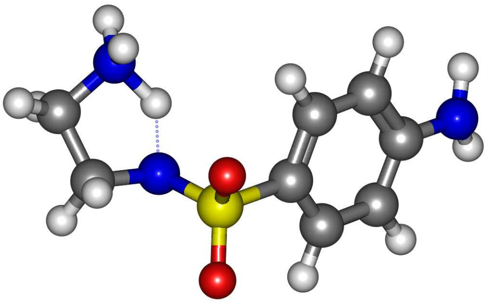	S	-0.892113	-0.941894	0.337312
	O	-1.295531	-0.667075	1.724442
	O	-0.966807	-2.361925	-0.014619
	N	4.697489	0.900017	-0.257636
	C	0.772371	-0.406009	0.152894
	C	1.174658	0.783152	0.750207
	C	1.672379	-1.145300	-0.600753
	C	3.391955	0.487531	-0.152781
	C	2.469868	1.227196	0.598877
	C	2.971054	-0.704769	-0.751203
	H	0.471261	1.363322	1.337071
	H	1.356613	-2.069017	-1.069610
	H	2.789238	2.153093	1.064462
	H	3.679759	-1.281276	-1.335380
	H	4.870391	1.879176	-0.085536
	H	5.222566	0.522541	-1.032145
	N	-1.663021	-0.020338	-0.710587
	C	-3.122911	-0.163631	-0.663500
	H	-3.474333	-0.624162	0.266787
	H	-3.508266	-0.755331	-1.494725
	C	-3.655687	1.269507	-0.743907
	H	-3.638891	1.627495	-1.772358
	H	-4.664205	1.360366	-0.349800
	N	-2.681787	2.069559	0.021693
	H	-2.903779	2.105740	1.012458
	H	-2.549791	3.007693	-0.336670
	H	-1.788934	1.281918	-0.193418
NethylS-closed/sulfonamide<br>E=-1025.3443845<br>G=-1025.169492	S	-0.733839	-1.065620	0.148079
	O	-1.024762	-1.435825	1.522696
	O	-0.929608	-2.095655	-0.854278
	N	4.742604	1.069362	-0.156545
	C	0.886642	-0.426814	0.067624
	C	1.555131	-0.067334	1.232190
	C	1.496507	-0.268140	-1.174624

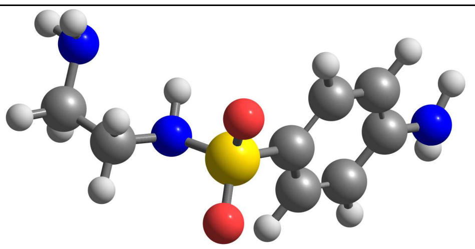	C	3.461794	0.606297	-0.083659
	C	2.830869	0.444308	1.157121
	C	2.769929	0.241883	-1.249742
	H	1.075952	-0.190599	2.195082
	H	0.970275	-0.546163	-2.080134
	H	3.360874	0.725796	2.059860
	H	3.254139	0.366269	-2.211647
	H	5.107733	1.554661	0.648309
	H	5.060707	1.415918	-1.048370
	N	-1.649554	0.224621	-0.342942
	C	-3.095855	-0.046410	-0.407110
	H	-3.455061	-0.529015	0.510857
	H	-3.291848	-0.717460	-1.243405
	C	-3.824255	1.257504	-0.604157
	H	-3.468051	1.736556	-1.519388
	H	-4.886574	1.029787	-0.750832
	N	-3.568844	2.154182	0.519236
	H	-4.057629	1.804010	1.337468
	H	-3.963983	3.065688	0.320986
	H	-1.484170	1.001401	0.300503
NethylS-open/sulfonamide<br>E=-1025.3426578<br>G=-1025.168097<br>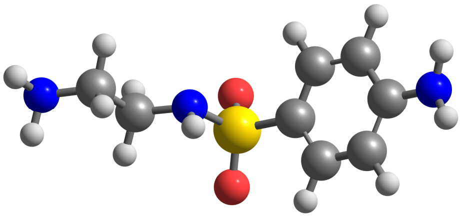	S	-0.617623	0.986407	0.246689
	O	-0.826479	2.026062	-0.746043
	O	-0.908001	1.336806	1.623019
	N	4.867989	-1.089059	-0.226415
	C	1.006973	0.373239	0.096019
	C	1.762336	0.666301	-1.033674
	C	1.531619	-0.424919	1.110033
	C	3.586333	-0.632649	-0.135789
	C	3.040352	0.168163	-1.148272
	C	2.807261	-0.921703	0.995812
	H	1.348894	1.285456	-1.819615
	H	0.937661	-0.654212	1.986887
	H	3.638200	0.392334	-2.024285
	H	3.225851	-1.541758	1.780331
	H	5.297854	-1.087375	-1.138469
	H	5.130325	-1.857504	0.371473
	N	-1.531105	-0.361323	-0.084987
	C	-2.982912	-0.129955	-0.041575
	H	-3.294960	0.613223	-0.785888
	H	-3.243050	0.253436	0.946355
	C	-3.697558	-1.433775	-0.293132
	H	-3.317219	-1.870441	-1.227441
	H	-3.455019	-2.136923	0.506820
	N	-5.137209	-1.207359	-0.304608
	H	-5.611449	-2.095832	-0.418300
	H	-5.375700	-0.657955	-1.124445
	H	-1.247729	-0.711913	-1.000714
NethylS^{-}<br>E=-1024.867589<br>G=-1024.706328	S	-0.950588	1.055299	0.030275
	O	-0.906204	2.385574	-0.598338
	O	-1.344392	1.098519	1.453687

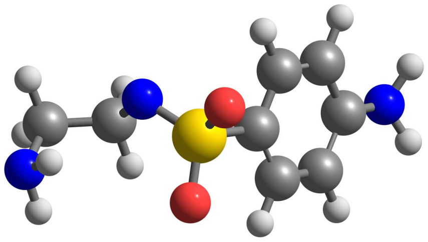

N	4.620124	-1.068919	-0.021110
C	0.734761	0.465636	0.063163
C	1.335341	0.016495	1.229332
C	1.444651	0.406613	-1.132110
C	3.349007	-0.530201	0.015090
C	2.628317	-0.476217	1.208816
C	2.735121	-0.078279	-1.158717
H	0.790746	0.056475	2.164401
H	0.983461	0.749398	-2.052072
H	3.094830	-0.823947	2.124498
H	3.287539	-0.115213	-2.091662
H	5.115782	-1.060801	0.858469
H	5.193419	-0.767946	-0.795700
N	-1.790961	0.109341	-0.866424
C	-1.875162	-1.267555	-0.394386
H	-1.532160	-1.390940	0.644095
H	-1.237624	-1.922179	-1.007746
C	-3.299822	-1.771618	-0.467515
H	-3.660469	-1.673731	-1.496471
H	-3.318394	-2.838356	-0.229004
N	-4.229422	-1.075765	0.419980
H	-4.092603	-0.079000	0.268721
H	-3.921826	-1.233133	1.376113

NpropylSH$_2^+$
E=-1065.5381509
G=-1065.308043

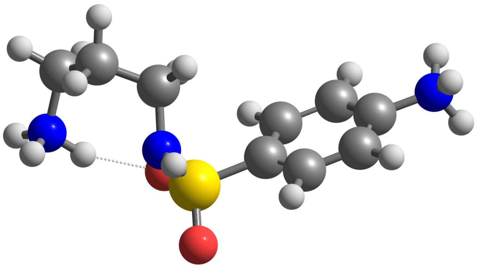

S	0.618981	1.134154	-0.160074
O	0.552566	2.527371	0.204982
O	1.239757	0.747414	-1.410957
N	-4.910773	-1.049707	-0.018965
C	-1.023225	0.472388	-0.148791
C	-1.848722	0.818296	0.911620
C	-1.440379	-0.381087	-1.154360
C	-3.543293	-0.533598	-0.057760
C	-3.129918	0.307421	0.957095
C	-2.726663	-0.892587	-1.107925
H	-1.499453	1.480577	1.694486
H	-0.776218	-0.642557	-1.967036
H	-3.800098	0.557242	1.770079
H	-3.085804	-1.561478	-1.880450
H	-5.212410	-1.228881	0.942985
H	-4.996465	-1.923207	-0.545739
N	1.462988	0.387428	1.022332
C	1.538834	-1.086947	1.006538
H	1.025953	-1.468643	0.118683
H	0.992698	-1.463165	1.871944
C	2.965226	-1.593830	1.029739
H	3.528051	-1.093273	1.824112
H	2.936632	-2.654646	1.290183
C	3.713722	-1.497772	-0.276383
H	4.674456	-2.002661	-0.202311
H	3.150450	-1.945343	-1.095588
N	4.004148	-0.087153	-0.679320

	H	4.677912	-0.064560	-1.444960
	H	4.395323	0.443277	0.101359
	H	3.150047	0.387433	-0.999739
	H	1.239751	0.787070	1.929343
	H	-5.571124	-0.379607	-0.425339
NpropylSH+ E=-1065.1035164 G=-1064.886757 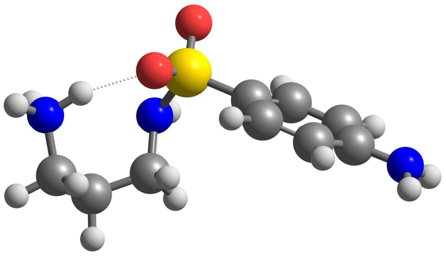	S	-0.566035	1.163104	0.165880
	O	-0.510109	2.563734	-0.188310
	O	-1.207670	0.775934	1.411846
	N	4.867555	-1.103458	0.042415
	C	1.044760	0.488130	0.158865
	C	1.891742	0.796817	-0.903763
	C	1.462042	-0.375805	1.164581
	C	3.597414	-0.607032	0.064692
	C	3.153715	0.256066	-0.950197
	C	2.728160	-0.914821	1.119636
	H	1.560429	1.461402	-1.693994
	H	0.796088	-0.621179	1.981976
	H	3.820390	0.492445	-1.771514
	H	3.063946	-1.584956	1.902692
	H	5.359838	-1.080060	-0.837330
	H	5.063452	-1.904155	0.623172
	N	-1.446594	0.450935	-1.028668
	C	-1.483251	-1.023089	-1.067888
	H	-0.941843	-1.424795	-0.205604
	H	-0.945592	-1.355487	-1.956593
	C	-2.896430	-1.568125	-1.080764
	H	-3.495075	-1.040514	-1.830243
	H	-2.850567	-2.612671	-1.398435
	C	-3.606098	-1.560376	0.250140
	H	-4.564116	-2.070867	0.176391
	H	-3.011772	-2.050599	1.021854
	N	-3.893386	-0.180034	0.746757
	H	-4.523463	-0.212595	1.548202
	H	-4.336049	0.384511	0.019705
	H	-3.027694	0.293988	1.043706
	H	-1.214522	0.875842	-1.921694
NpropylS-open/amine E=-1064.6240772 G= -1064.422248 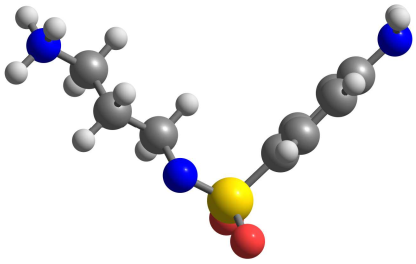	S	-0.015549	1.806708	-0.002291
	O	0.450194	2.844844	-0.933186
	O	-0.102151	2.263003	1.399177
	N	4.169828	-2.437295	-0.044884
	C	1.260307	0.559069	0.019651
	C	1.768476	0.095767	-1.189648
	C	1.703447	-0.011852	1.203383
	C	3.179288	-1.476120	-0.023301
	C	2.719084	-0.902602	-1.214377
	C	2.652369	-1.018712	1.185471
	H	1.418050	0.523394	-2.122662
	H	1.306302	0.334667	2.149584
	H	3.119590	-1.254272	-2.159288
	H	2.996632	-1.461443	2.114128

	H	4.256985	-2.948735	-0.910913
	H	4.211948	-3.033072	0.769067
	N	-1.321273	1.180841	-0.570810
	C	-1.895129	0.150202	0.276411
	H	-2.211181	0.542707	1.254837
	H	-1.176631	-0.659372	0.494758
	C	-3.098956	-0.458425	-0.407915
	H	-3.832572	0.324813	-0.629440
	H	-2.796964	-0.899392	-1.364470
	C	-3.733450	-1.513560	0.457500
	H	-3.044522	-2.328559	0.677438
	H	-4.095366	-1.103269	1.399831
	N	-4.909718	-2.112709	-0.237116
	H	-5.603484	-1.394737	-0.454183
	H	-5.362388	-2.826081	0.335058
	H	-4.626448	-2.545227	-1.118390
NpropylS-closed/amine<br>E=-1064.6292124<br>G=-1064.425148<br>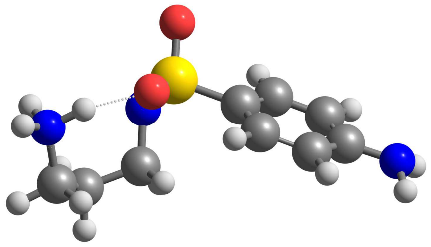	S	-0.655766	1.083697	0.023351
	O	-0.555245	2.522338	-0.247269
	O	-1.210691	0.793148	1.374876
	N	4.921193	-0.990471	0.113559
	C	1.009775	0.460268	0.098623
	C	1.835779	0.654602	-1.004078
	C	1.486611	-0.248262	1.191055
	C	3.617186	-0.544183	0.095244
	C	3.124178	0.165245	-1.006682
	C	2.776683	-0.746258	1.191720
	H	1.466773	1.195809	-1.868645
	H	0.845420	-0.412921	2.047797
	H	3.768407	0.322047	-1.865169
	H	3.147197	-1.301083	2.047048
	H	5.351668	-1.113591	-0.791101
	H	5.112555	-1.749742	0.750606
	N	-1.424508	0.400391	-1.134566
	C	-1.503328	-1.058765	-1.059665
	H	-0.950708	-1.481199	-0.207245
	H	-1.029550	-1.479656	-1.954085
	C	-2.928969	-1.579733	-1.003137
	H	-3.537998	-1.071184	-1.759092
	H	-2.924223	-2.641048	-1.268972
	C	-3.606514	-1.488099	0.341511
	H	-4.583908	-1.967039	0.316971
	H	-3.009929	-1.965553	1.120227
	N	-3.824464	-0.077920	0.779860
	H	-4.439274	-0.043175	1.591956
	H	-4.253387	0.469842	0.033350
	H	-2.917261	0.362572	1.032370
NpropylS-closed/TS<br>E=-1064.6253605<br>G=-1064.426594	S	-0.525918	1.171380	0.148483
	O	-0.605839	2.298483	-0.779786
	O	-0.702950	1.524920	1.560382
	N	4.877429	-1.229121	-0.335224

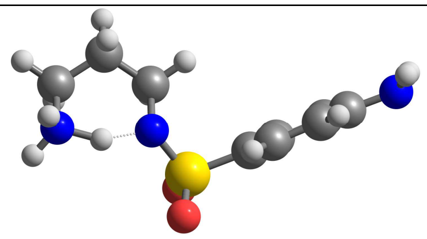

C	1.094840	0.474059	0.027421
C	1.642399	0.248912	-1.231512
C	1.798694	0.096493	1.161742
C	3.612763	-0.702289	-0.214881
C	2.887267	-0.327618	-1.353484
C	3.045938	-0.485192	1.043905
H	1.090627	0.529351	-2.121768
H	1.369313	0.259360	2.142430
H	3.318901	-0.497111	-2.333930
H	3.597669	-0.778975	1.930191
H	5.095513	-1.653417	-1.224521
H	5.212032	-1.757761	0.456675
N	-1.532071	0.064682	-0.369005
C	-1.598269	-1.157805	0.442804
H	-1.618447	-0.930611	1.517777
H	-0.722732	-1.795115	0.264840
C	-2.866256	-1.911627	0.084403
H	-2.821445	-2.245576	-0.958594
H	-2.942780	-2.806123	0.705335
C	-4.101454	-1.061120	0.286943
H	-5.020391	-1.623689	0.123906
H	-4.122986	-0.662509	1.303399
N	-4.037386	0.100008	-0.636872
H	-4.648386	0.855827	-0.344693
H	-4.292004	-0.176435	-1.581609
H	-2.861215	0.361625	-0.596267

NpropylS-closed/sulfonamide
E=-1064.6387428
G= -1064.436922

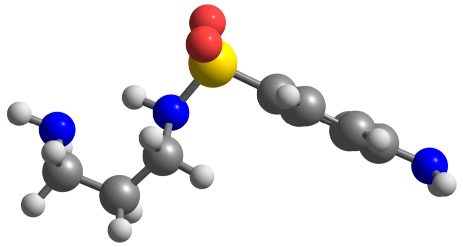

S	0.451299	1.297323	-0.102956
O	0.574816	2.297863	0.940982
O	0.659865	1.738178	-1.471772
N	-4.844836	-1.270329	0.220627
C	-1.117330	0.529210	-0.022649
C	-1.662257	0.227384	1.222807
C	-1.805046	0.207061	-1.185940
C	-3.602437	-0.708100	0.137572
C	-2.891409	-0.382133	1.302715
C	-3.036278	-0.406161	-1.107202
H	-1.121179	0.472317	2.129421
H	-1.373929	0.436641	-2.152213
H	-3.325092	-0.616495	2.268357
H	-3.579324	-0.660486	-2.010361
H	-5.088413	-1.718191	1.090817
H	-5.196262	-1.731099	-0.604872
N	1.488512	0.096352	0.323578
C	1.691993	-0.970668	-0.667674
H	1.978085	-0.555768	-1.641627
H	0.743564	-1.498952	-0.798475
C	2.763549	-1.929347	-0.194909
H	2.506738	-2.312968	0.799617
H	2.762862	-2.787352	-0.872314
C	4.157706	-1.336599	-0.172200

H	4.886491	-2.145945	-0.040242
H	4.367727	-0.880074	-1.144585
N	4.288457	-0.296338	0.854950
H	5.216799	0.107875	0.808007
H	4.217373	-0.733585	1.769141
H	2.390453	0.486900	0.623737

NpropylS-open/sulfonamide
E=-1064.6377922
G=-1064.436803

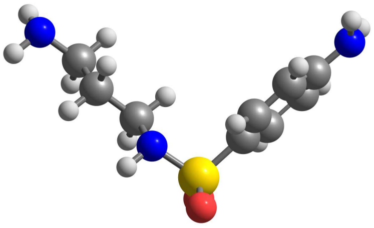

S	0.014902	1.781532	0.047034
O	0.338207	2.826275	-0.905579
O	-0.202306	2.175968	1.426802
N	4.195566	-2.366480	-0.061590
C	1.239789	0.537001	0.027643
C	1.758666	0.113541	-1.193815
C	1.691064	-0.024805	1.215661
C	3.199578	-1.433692	-0.032927
C	2.727636	-0.859964	-1.224319
C	2.662085	-1.000834	1.186047
H	1.401258	0.548509	-2.119957
H	1.280243	0.303766	2.161778
H	3.138769	-1.194669	-2.169915
H	3.019900	-1.444250	2.108302
H	4.363131	-2.837135	-0.937540
H	4.316037	-2.938308	0.760178
N	-1.309907	1.024268	-0.588261
C	-1.995557	0.076501	0.297885
H	-2.397006	0.580626	1.183683
H	-1.259517	-0.657061	0.643164
C	-3.103215	-0.618396	-0.456604
H	-3.800200	0.133012	-0.849666
H	-2.687954	-1.150479	-1.318414
C	-3.854286	-1.583226	0.429685
H	-3.166380	-2.352813	0.792720
H	-4.215095	-1.045330	1.317805
N	-4.928960	-2.236599	-0.313691
H	-5.601321	-1.529258	-0.594959
H	-5.431261	-2.861359	0.307132
H	-1.937697	1.737209	-0.957372

NpropylS^{-}
E=-1064.1571788
G=-1063.968873

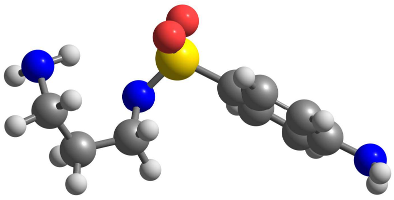

S	-0.569963	1.096886	-0.004459
O	-0.386579	2.442660	-0.572534
O	-1.112221	1.118370	1.370369
N	4.890072	-1.249910	0.468311
C	1.067069	0.409104	0.181349
C	1.898766	0.357835	-0.932808
C	1.508276	-0.128782	1.380668
C	3.612133	-0.737642	0.365092
C	3.156223	-0.201090	-0.844622
C	2.766328	-0.697323	1.474420
H	1.558506	0.761918	-1.880204
H	0.863408	-0.103604	2.250284
H	3.803828	-0.232991	-1.714408
H	3.107116	-1.117022	2.414961

	H	5.303369	-1.558117	-0.399754
	H	5.033022	-1.903532	1.224327
	N	-1.368019	0.235295	-1.016823
	C	-1.561224	-1.157365	-0.611469
	H	-1.334412	-1.324543	0.452585
	H	-0.867242	-1.803167	-1.169498
	C	-2.978180	-1.645300	-0.849749
	H	-3.257326	-1.488227	-1.899587
	H	-2.991860	-2.728438	-0.679665
	C	-4.015438	-0.998048	0.052542
	H	-4.932125	-1.593404	0.043041
	H	-3.647665	-1.025418	1.085005
	N	-4.371865	0.385860	-0.271109
	H	-4.808184	0.384856	-1.188791
	H	-3.495674	0.886213	-0.404823
NbutylSH$_2^+$<br>E=-1104.8300869<br>G=-1104.571253<br>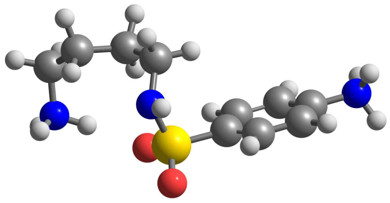	S	0.338613	1.255800	-0.192329
	O	0.171036	2.687265	-0.118625
	O	1.093027	0.691711	-1.290344
	N	-5.029965	-1.304392	-0.115553
	C	-1.261210	0.496132	-0.210467
	C	-2.251078	1.041477	0.595160
	C	-1.478895	-0.639585	-0.969776
	C	-3.706645	-0.683381	-0.144304
	C	-3.494657	0.443976	0.626260
	C	-2.727506	-1.238753	-0.937919
	H	-2.057198	1.925055	1.190598
	H	-0.689618	-1.053076	-1.583401
	H	-4.291199	0.845505	1.240454
	H	-2.931954	-2.127491	-1.522310
	H	-5.368367	-1.412285	0.844941
	H	-5.016649	-2.230576	-0.551010
	N	1.061423	0.815302	1.215181
	C	2.022993	-1.452182	0.793893
	H	1.715436	-1.433550	-0.255825
	H	1.895272	-2.489421	1.112798
	C	3.487295	-1.044617	0.945627
	H	3.566499	0.002408	1.258281
	H	3.955310	-1.630928	1.738547
	C	4.294154	-1.227420	-0.314895
	H	5.365075	-1.198888	-0.125483
	H	4.055327	-2.161436	-0.822951
	N	4.005539	-0.116705	-1.275481
	H	4.444432	-0.281655	-2.181081
	H	4.368627	0.763555	-0.904571
	H	2.995818	0.013187	-1.421654
	H	0.710880	1.419666	1.954343
	H	-5.719012	-0.735629	-0.617781
	C	1.075254	-0.607094	1.613308
	H	0.064250	-1.028000	1.586113
	H	1.389555	-0.601799	2.658649

NbutylSH$^+$
E=-1104.3952494
G=-1104.150119

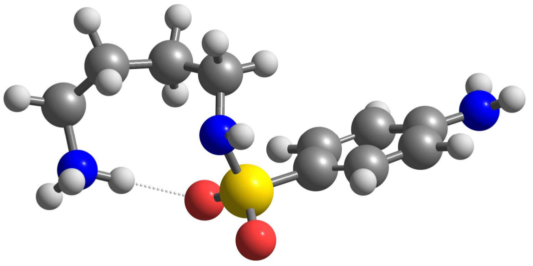

S	0.303448	1.210734	-0.121924
O	0.204968	2.632265	0.127229
O	1.033031	0.761544	-1.295367
N	-5.066305	-1.201836	-0.188506
C	-1.293307	0.508919	-0.187339
C	-2.263440	0.970196	0.699844
C	-1.570093	-0.549308	-1.046103
C	-3.813580	-0.663287	-0.164529
C	-3.510417	0.393483	0.708548
C	-2.819998	-1.126371	-1.037943
H	-2.039640	1.782776	1.381550
H	-0.807332	-0.916298	-1.721542
H	-4.273884	0.750857	1.390006
H	-3.047108	-1.947204	-1.708643
H	-5.658293	-1.035085	0.610555
H	-5.174719	-2.105642	-0.622257
N	1.088096	0.599870	1.201913
C	1.977873	-1.644775	0.556727
H	1.712279	-1.494640	-0.494336
H	1.787613	-2.702395	0.752306
C	3.458301	-1.332203	0.799946
H	3.565255	-0.479341	1.479361
H	3.938192	-2.174005	1.302001
C	4.250642	-1.049394	-0.451212
H	5.321588	-1.046938	-0.257723
H	4.042638	-1.773069	-1.238810
N	3.916726	0.300520	-1.000475
H	4.418052	0.477914	-1.870713
H	4.182740	1.026377	-0.332390
H	2.906604	0.406710	-1.184154
H	0.767621	1.123603	2.012703
C	1.035071	-0.855602	1.437263
H	0.010465	-1.230148	1.331233
H	1.312832	-0.985501	2.484990

NbutylS-open/amine
E=-1103.9153808
G=-1103.685710

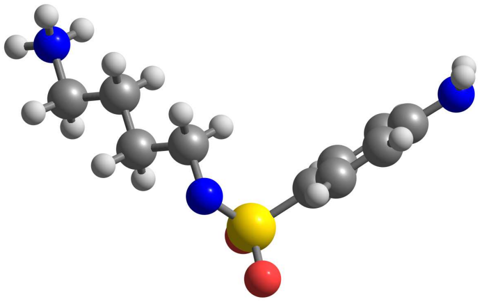

S	0.403893	1.681480	0.220320
O	1.090651	2.966920	0.015489
O	-0.171821	1.533508	1.573410
N	4.676528	-2.466597	-0.122162
C	1.683438	0.435005	0.158697
C	2.530853	0.399551	-0.944235
C	1.824268	-0.523670	1.150253
C	3.662406	-1.532483	-0.045797
C	3.511145	-0.564619	-1.045732
C	2.800681	-1.499439	1.051113
H	2.422766	1.136567	-1.732704
H	1.165380	-0.508326	2.009497
H	4.174069	-0.584213	-1.904379
H	2.905232	-2.248567	1.828856
H	5.023151	-2.650198	-1.052454
H	4.527803	-3.309042	0.413909

N	-0.581522	1.452945	-0.957971
C	-2.761516	0.413690	-0.352014
H	-3.272996	1.153309	-0.979557
H	-2.728700	0.830897	0.660436
C	-3.547465	-0.882853	-0.348665
H	-3.568404	-1.309720	-1.358752
H	-3.053553	-1.618934	0.297100
C	-4.957228	-0.664580	0.129553
H	-4.984735	-0.277544	1.147694
H	-5.501434	0.023323	-0.516828
N	-5.716080	-1.950615	0.132578
H	-5.273810	-2.628675	0.755654
H	-6.679687	-1.819152	0.440859
C	-1.349102	0.216664	-0.867763
H	-0.856988	-0.551214	-0.249111
H	-1.412409	-0.220552	-1.872207
H	-5.735980	-2.358785	-0.803899

NbutylS-closed/amine
E=-1103.9227496
G= -1103.693671

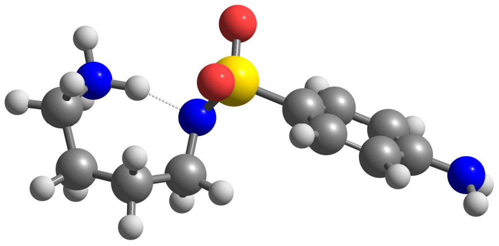

S	0.327967	-0.799632	0.543519
O	0.400558	-2.264669	0.462835
O	0.630562	-0.259154	1.879168
N	-5.336457	0.652251	-0.555335
C	-1.368308	-0.354713	0.247279
C	-2.009738	-0.864722	-0.877298
C	-2.050233	0.515397	1.084492
C	-4.014566	0.350823	-0.308371
C	-3.315931	-0.522750	-1.151226
C	-3.359545	0.866984	0.811273
H	-1.481568	-1.537234	-1.544217
H	-1.554984	0.921015	1.957781
H	-3.816096	-0.926419	-2.025027
H	-3.889559	1.549118	1.467245
H	-5.638514	0.536559	-1.511522
H	-5.676890	1.502227	-0.130351
N	1.193558	-0.219273	-0.624311
C	2.485997	1.825946	0.110115
H	2.394802	1.574455	1.171844
H	2.398927	2.915759	0.041478
C	3.877995	1.439849	-0.379349
H	3.946022	1.561412	-1.468572
H	4.597517	2.144456	0.045818
C	4.367446	0.058240	-0.001440
H	5.449999	-0.011665	-0.094061
H	4.096423	-0.188900	1.026684
N	3.778763	-0.995536	-0.870760
H	4.081577	-0.870742	-1.836160
H	4.057225	-1.927295	-0.568994
C	1.313467	1.238285	-0.661327
H	0.391139	1.728104	-0.316514
H	1.421766	1.525759	-1.714382
H	2.715217	-0.894039	-0.825437

NbutylS-closed/TS
E=-1103.9212741
G=-1103.694770

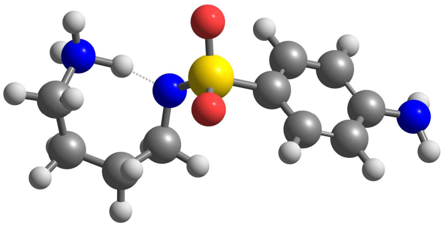

S	0.323007	-0.740129	0.601680
O	0.417602	-2.201919	0.565267
O	0.628025	-0.138177	1.904703
N	-5.320474	0.661226	-0.600824
C	-1.362192	-0.307125	0.266944
C	-2.002479	-0.885759	-0.824629
C	-2.040059	0.615906	1.049537
C	-4.002419	0.370942	-0.333682
C	-3.306394	-0.556449	-1.120336
C	-3.346897	0.953972	0.753257
H	-1.475809	-1.601095	-1.446462
H	-1.543756	1.072534	1.896584
H	-3.807648	-1.012008	-1.967429
H	-3.875525	1.677080	1.364741
H	-5.627795	0.482366	-1.545373
H	-5.665480	1.531208	-0.222961
N	1.194684	-0.196841	-0.596737
C	2.594763	1.789278	0.068763
H	2.510231	1.568632	1.138005
H	2.574995	2.880153	-0.026430
C	3.945097	1.294725	-0.440261
H	4.003100	1.410386	-1.530851
H	4.725053	1.939681	-0.026908
C	4.316930	-0.127088	-0.072255
H	5.386710	-0.290370	-0.203757
H	4.072965	-0.330398	0.973361
N	3.578958	-1.113562	-0.893028
H	3.857044	-1.039542	-1.869285
H	3.761852	-2.064550	-0.584406
C	1.373524	1.255262	-0.663207
H	0.480530	1.779642	-0.299257
H	1.461919	1.519836	-1.723498
H	2.437049	-0.838471	-0.783379

NbutylS-closed/sulfonamide
E=-1103.9305436
G=-1103.701285

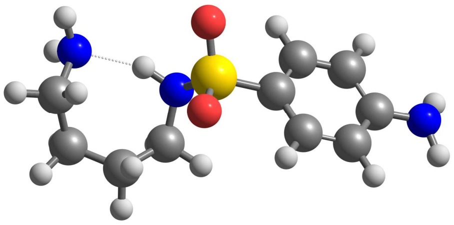

S	0.293230	-0.694605	0.656245
O	0.473166	-2.137321	0.676133
O	0.636276	0.027484	1.870634
N	-5.338514	0.531689	-0.613533
C	-1.373183	-0.304974	0.278257
C	-2.022284	-1.009756	-0.732168
C	-2.041885	0.694110	0.973132
C	-4.022319	0.280247	-0.341153
C	-3.331547	-0.723182	-1.037456
C	-3.353732	0.985562	0.666003
H	-1.498757	-1.785406	-1.279200
H	-1.533441	1.243647	1.755033
H	-3.843883	-1.270478	-1.820673
H	-3.880302	1.765904	1.203800
H	-5.689649	0.216313	-1.504865
H	-5.708572	1.417460	-0.304095
N	1.130115	-0.188097	-0.649532

	C	2.555505	1.811554	-0.037645
	H	2.408697	1.655619	1.035530
	H	2.535261	2.894926	-0.196296
	C	3.936419	1.301491	-0.437256
	H	4.061264	1.370725	-1.526442
	H	4.672423	1.988039	-0.008931
	C	4.311249	-0.101339	0.014746
	H	5.401548	-0.161632	0.110688
	H	3.898924	-0.287976	1.012757
	N	3.798490	-1.137547	-0.889735
	H	4.219658	-1.009784	-1.804697
	H	4.093784	-2.050886	-0.562797
	C	1.373580	1.250044	-0.804599
	H	0.459136	1.783056	-0.527403
	H	1.516838	1.426416	-1.875129
	H	1.998395	-0.748424	-0.762900
NbutylS-open/sulfonamide<br>E=-1103.9296771<br>G= -1103.701940<br>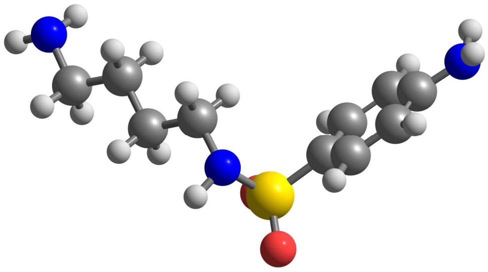	S	-0.352159	1.535808	-0.346484
	O	-0.829737	2.891674	-0.148218
	O	0.331595	1.242023	-1.592427
	N	-4.869914	-2.211424	0.181942
	C	-1.669738	0.397805	-0.185259
	C	-2.589831	0.568870	0.846495
	C	-1.805313	-0.657603	-1.078205
	C	-3.796951	-1.373106	0.079780
	C	-3.641799	-0.304957	0.977371
	C	-2.858502	-1.535956	-0.946574
	H	-2.480074	1.388882	1.546811
	H	-1.084606	-0.789753	-1.875099
	H	-4.364101	-0.177117	1.775702
	H	-2.970048	-2.362591	-1.638970
	H	-5.354688	-2.233980	1.065947
	H	-4.807333	-3.099623	-0.291438
	N	0.586417	1.228236	0.972222
	C	2.826318	0.298019	0.381029
	H	3.299556	1.088745	0.977005
	H	2.751530	0.678773	-0.642840
	C	3.686663	-0.948852	0.410809
	H	3.734588	-1.344596	1.432538
	H	3.220638	-1.731105	-0.203122
	C	5.086321	-0.691707	-0.093110
	H	5.025710	-0.237815	-1.092924
	H	5.573040	0.046964	0.551283
	N	5.883654	-1.916829	-0.075265
	H	5.465245	-2.580630	-0.720102
	H	6.807484	-1.720751	-0.444521
	C	1.442730	0.037025	0.931483
	H	0.929394	-0.733750	0.347595
	H	1.513757	-0.350386	1.951485
	H	1.079091	2.079120	1.234349
NbutylS^{-}	S	-0.428410	1.124648	0.236965

E=-1103.4466129<br>G=-1103.230784<br>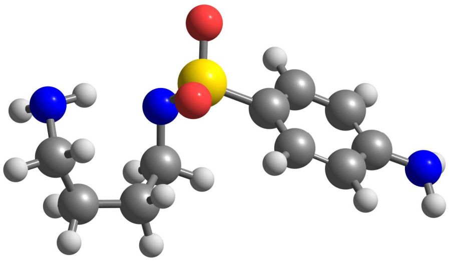	O	-0.335384	2.587460	0.092349
	O	-0.934848	0.729066	1.568456
	N	5.117622	-1.019988	-0.097474
	C	1.239439	0.499150	0.189353
	C	2.092206	0.933178	-0.820055
	C	1.676790	-0.473136	1.076446
	C	3.822905	-0.548181	-0.025729
	C	3.369351	0.423894	-0.924938
	C	2.954887	-0.992056	0.973556
	H	1.752814	1.679225	-1.530250
	H	1.013295	-0.827385	1.856028
	H	4.035451	0.770858	-1.707730
	H	3.294803	-1.750979	1.670226
	H	5.560677	-0.932718	-1.000359
	H	5.274177	-1.919624	0.332922
	N	-1.206568	0.564071	-0.986645
	C	-1.908865	-1.794945	-0.204496
	H	-1.551043	-1.607707	0.814108
	H	-1.582821	-2.811655	-0.454985
	C	-3.434784	-1.789568	-0.218512
	H	-3.794172	-1.844903	-1.255923
	H	-3.771102	-2.713967	0.266159
	C	-4.131150	-0.633628	0.482268
	H	-5.164467	-0.923893	0.692811
	H	-3.659476	-0.473642	1.458432
	N	-4.161375	0.642065	-0.231369
	H	-4.658697	0.496501	-1.105389
	H	-3.200313	0.860926	-0.501859
	C	-1.184043	-0.880671	-1.188546
	H	-0.143913	-1.238913	-1.258729
	H	-1.616978	-1.055959	-2.180995
NpentylSH$_2^+$<br>E=-1144.1213357<br>G=-1143.835784<br>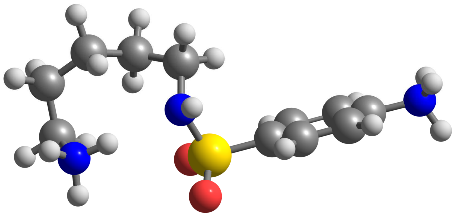	S	0.061572	0.931335	-0.238139
	O	0.150395	2.295029	0.227556
	O	0.561382	0.584684	-1.545097
	N	-5.626628	-0.774316	0.119114
	C	-1.631713	0.418032	-0.145835
	C	-2.374708	0.793543	0.964183
	C	-2.169416	-0.356662	-1.158684
	C	-4.223382	-0.373236	0.035076
	C	-3.692640	0.391631	1.055611
	C	-3.491012	-0.757915	-1.066324
	H	-1.935214	1.394785	1.750639
	H	-1.570143	-0.640818	-2.013043
	H	-4.301076	0.668250	1.907662
	H	-3.943765	-1.363753	-1.841592
	H	-5.914981	-0.918791	1.090762
	H	-5.799604	-1.642998	-0.393903
	N	0.900807	0.035401	0.861246
	C	2.191559	-1.883508	-0.042459
	H	2.217946	-1.423017	-1.033925

	H	2.108510	-2.958914	-0.217264
	C	3.467194	-1.570164	0.743131
	H	3.232991	-0.909127	1.585713
	H	3.851379	-2.482325	1.204576
	C	4.232655	0.372346	-0.725855
	H	5.095099	0.811109	-1.222937
	H	3.428274	0.286904	-1.456168
	N	3.768344	1.361535	0.295049
	H	3.622015	2.280178	-0.124387
	H	4.454494	1.460549	1.046267
	H	2.879308	1.058359	0.715039
	H	0.618638	0.312844	1.798721
	H	-6.241356	-0.056416	-0.277082
	C	0.934131	-1.434633	0.668151
	H	0.040502	-1.770427	0.131952
	H	0.883917	-1.869796	1.667744
	C	4.582929	-0.948759	-0.084540
	H	5.469678	-0.812186	0.542657
	H	4.872771	-1.625883	-0.893459
NpentylSH$^+$ E=-1143.6866228 G= -1143.414600 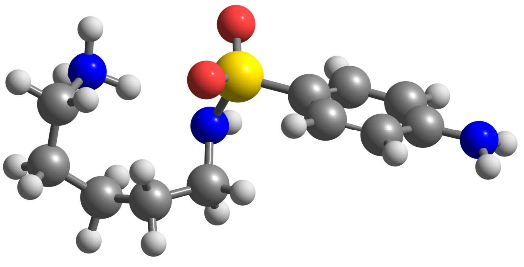	S	-0.006689	-0.932882	-0.240856
	O	-0.112181	-2.301662	0.218777
	O	-0.540866	-0.580136	-1.536708
	N	5.613488	0.790565	0.103608
	C	1.660735	-0.420604	-0.161350
	C	2.440059	-0.829363	0.919080
	C	2.192674	0.415069	-1.137466
	C	4.303242	0.419539	0.031816
	C	3.746437	-0.415217	1.013722
	C	3.502479	0.828123	-1.043184
	H	2.021341	-1.473623	1.683951
	H	1.581262	0.735092	-1.971704
	H	4.362037	-0.731329	1.848067
	H	3.927324	1.475222	-1.802129
	H	6.072341	0.702440	0.997162
	H	5.904205	1.579161	-0.453467
	N	-0.875215	-0.059240	0.877626
	C	-2.149830	1.877653	-0.011826
	H	-2.169996	1.429828	-1.009243
	H	-2.059608	2.954706	-0.172520
	C	-3.434588	1.562093	0.759156
	H	-3.207545	0.902551	1.604880
	H	-3.826265	2.473727	1.215469
	C	-4.177197	-0.370992	-0.737726
	H	-5.042672	-0.827813	-1.212932
	H	-3.398338	-0.257734	-1.491506
	N	-3.654079	-1.353604	0.260181
	H	-3.500833	-2.266179	-0.169552
	H	-4.309997	-1.472615	1.034956
	H	-2.754580	-1.027591	0.648459
	H	-0.559725	-0.336608	1.804670

	C	-0.901547	1.412164	0.706077
	H	-0.000192	1.753869	0.185825
	H	-0.863219	1.835815	1.711474
	C	-4.542910	0.936313	-0.075735
	H	-5.424488	0.778507	0.553777
	H	-4.846329	1.620341	-0.873801
NpentylS-open/amine<br>E=-1143.2072941<br>G=-1142.951723<br>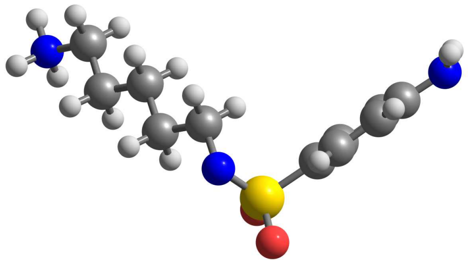	S	0.889819	1.749240	0.305426
	O	1.658073	3.004035	0.254865
	O	0.183814	1.546547	1.587943
	N	4.957211	-2.608905	0.072420
	C	2.104721	0.438019	0.274641
	C	3.055631	0.427664	-0.741069
	C	2.094396	-0.593465	1.200832
	C	3.989350	-1.624573	0.118560
	C	3.989892	-0.583979	-0.817491
	C	3.024143	-1.616063	1.125945
	H	3.066264	1.221548	-1.479855
	H	1.353734	-0.598580	1.990799
	H	4.734199	-0.584160	-1.606745
	H	3.010701	-2.421629	1.852464
	H	5.382933	-2.752373	-0.831350
	H	4.714465	-3.473695	0.533571
	N	0.009201	1.653704	-0.970051
	C	-2.244943	0.662443	-0.547218
	H	-2.702494	1.486875	-1.108218
	H	-2.227526	0.973110	0.503432
	C	-3.089714	-0.587009	-0.697397
	H	-3.117950	-0.889936	-1.751867
	H	-2.618935	-1.416256	-0.154171
	C	-5.316295	-1.655166	-0.347720
	H	-5.417556	-1.943609	-1.393625
	H	-4.879223	-2.489503	0.200292
	N	-6.696490	-1.459936	0.186819
	H	-7.263353	-2.301587	0.081712
	H	-7.167143	-0.696229	-0.302486
	C	-0.824119	0.457518	-1.035469
	H	-0.387962	-0.394887	-0.488633
	H	-0.866558	0.133597	-2.082903
	C	-4.505155	-0.397527	-0.189048
	H	-4.483980	-0.110010	0.868921
	H	-4.992176	0.421620	-0.731399
	H	-6.666535	-1.218008	1.179070
NpentylS-closed/amine<br>E=-1143.2144892<br>G=-1142.955657	S	0.081359	-0.692076	0.475436
	O	0.206522	-2.138524	0.247226
	O	0.358219	-0.276461	1.860868
	N	-5.637652	0.636830	-0.501294
	C	-1.632640	-0.287765	0.218440
	C	-2.249658	-0.700617	-0.958548
	C	-2.352450	0.459979	1.138001
	C	-4.304457	0.365012	-0.279023

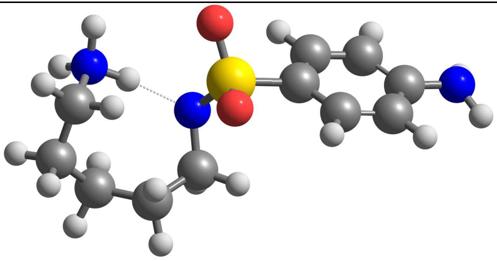

C	-3.568100	-0.384996	-1.204558
C	-3.674238	0.785728	0.893364
H	-1.692301	-1.275845	-1.689776
H	-1.877785	0.789816	2.053514
H	-4.048293	-0.713202	-2.120246
H	-4.234184	1.371829	1.614178
H	-5.931637	0.611599	-1.466701
H	-6.011147	1.425381	0.006284
N	0.932964	0.031330	-0.617645
C	2.412248	1.886329	0.156760
H	2.506369	1.461561	1.161881
H	2.405076	2.972968	0.286790
C	3.608234	1.476116	-0.711890
H	3.247013	1.085529	-1.670820
H	4.196584	2.359382	-0.971992
C	3.961963	-0.849910	0.330427
H	4.731975	-1.534756	0.680913
H	3.221759	-0.736346	1.124451
N	3.250099	-1.491865	-0.808754
H	3.031476	-2.465549	-0.603993
H	3.807911	-1.458836	-1.662208
H	2.335480	-0.968278	-0.937139
C	1.072452	1.477568	-0.434126
H	0.263911	1.896107	0.181260
H	0.971728	1.951824	-1.418140
C	4.565634	0.472676	-0.080143
H	5.389099	0.276906	-0.775881
H	5.015492	0.908218	0.817523

NpentylS-closed/TS
E=-1143.2119053
G=-1142.957780

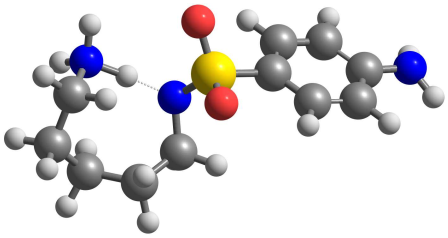

S	0.093027	-0.611143	0.657146
O	0.293864	-2.062595	0.667034
O	0.319901	0.052578	1.945199
N	-5.604472	0.335039	-0.722419
C	-1.609885	-0.311596	0.269618
C	-2.167738	-0.941092	-0.839073
C	-2.383716	0.553756	1.028468
C	-4.276313	0.150586	-0.414159
C	-3.483985	-0.717932	-1.176338
C	-3.703741	0.784578	0.691046
H	-1.566010	-1.611471	-1.442685
H	-1.952109	1.050149	1.888363
H	-3.920297	-1.211692	-2.037781
H	-4.307990	1.462824	1.283674
H	-5.866844	0.134077	-1.676042
H	-6.028084	1.175271	-0.357199
N	0.959444	-0.041228	-0.534034
C	2.368338	1.978302	-0.074415
H	2.481201	1.715527	0.983091
H	2.295496	3.069411	-0.109810
C	3.593534	1.519602	-0.875541
H	3.262788	1.062795	-1.816290

	H	4.180862	2.390298	-1.177389
	C	3.934414	-0.711676	0.365134
	H	4.713176	-1.374783	0.742182
	H	3.239047	-0.519750	1.187098
	N	3.156939	-1.396343	-0.690125
	H	2.967926	-2.362417	-0.437115
	H	3.652437	-1.388447	-1.579028
	H	2.118012	-0.809572	-0.729011
	C	1.055216	1.420548	-0.598547
	H	0.220049	1.893693	-0.067353
	H	0.947457	1.717502	-1.648290
	C	4.543817	0.571723	-0.153254
	H	5.365856	0.316691	-0.831545
	H	4.995456	1.085248	0.701339
NpentylS-closed/sulfonamide<br>E=-1143.2226889<br>G=-1142.965696<br>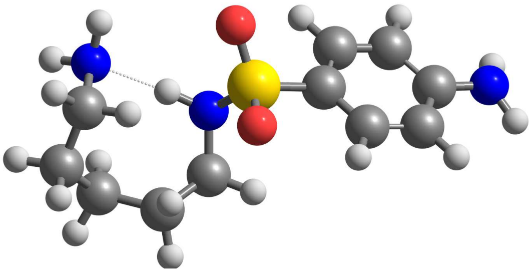	S	0.054413	-0.686006	0.557037
	O	0.280072	-2.110852	0.385319
	O	0.381694	-0.116589	1.853869
	N	-5.626909	0.488268	-0.523212
	C	-1.625361	-0.304423	0.235092
	C	-2.246287	-0.865858	-0.877900
	C	-2.334673	0.539158	1.079671
	C	-4.299703	0.254884	-0.290949
	C	-3.567732	-0.591367	-1.137917
	C	-3.659270	0.817632	0.819096
	H	-1.691387	-1.519320	-1.541342
	H	-1.848014	0.977986	1.941309
	H	-4.058277	-1.026597	-2.001393
	H	-4.218131	1.476624	1.473930
	H	-5.966363	0.308151	-1.455699
	H	-6.033535	1.295578	-0.075866
	N	0.868347	0.025507	-0.669379
	C	2.345946	1.925935	0.045348
	H	2.395863	1.541635	1.069233
	H	2.303707	3.014981	0.133094
	C	3.595976	1.515619	-0.746290
	H	3.298290	1.073341	-1.703593
	H	4.167605	2.409400	-1.008853
	C	3.949702	-0.787815	0.342412
	H	4.719912	-1.374901	0.857380
	H	3.130443	-0.666497	1.060584
	N	3.421507	-1.475345	-0.839018
	H	3.182785	-2.431098	-0.598373
	H	4.146971	-1.524296	-1.547815
	H	1.727424	-0.515476	-0.877913
	C	1.046527	1.482640	-0.596895
	H	0.188021	1.905703	-0.067621
	H	0.994214	1.866472	-1.619734
	C	4.534933	0.561172	-0.020764
	H	5.420079	0.394946	-0.647103
	H	4.894694	1.034508	0.899512

NpentylS-open/sulfonamide
E=-1143.2216779
G=-1142.969509

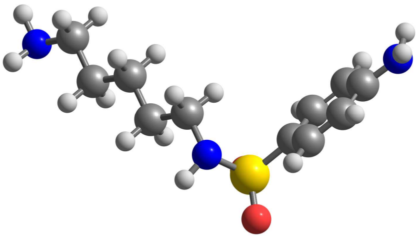

S	0.925658	1.617827	0.349681
O	1.535988	2.920630	0.159889
O	0.206446	1.391829	1.589758
N	5.033007	-2.579235	-0.154013
C	2.123439	0.353196	0.195999
C	3.055838	0.420762	-0.836534
C	2.150513	-0.703353	1.097428
C	4.055121	-1.631027	-0.056103
C	4.011363	-0.558660	-0.960975
C	3.106871	-1.686770	0.972646
H	3.030554	1.242474	-1.542975
H	1.420831	-0.754439	1.895409
H	4.741541	-0.513279	-1.761079
H	3.132846	-2.515179	1.671401
H	5.509807	-2.658336	-1.039106
H	4.874798	-3.453599	0.322986
N	-0.025206	1.406280	-0.978664
C	-2.324649	0.601235	-0.406799
H	-2.742380	1.452501	-0.959032
H	-2.230117	0.919367	0.636731
C	-3.266297	-0.582397	-0.505545
H	-3.341939	-0.900538	-1.553274
H	-2.839780	-1.433790	0.040682
C	-5.583182	-1.462043	-0.066263
H	-5.605536	-1.809947	-1.109430
H	-5.182522	-2.290920	0.525783
N	-6.911617	-1.128574	0.446197
H	-7.525973	-1.927053	0.330787
H	-7.303690	-0.394504	-0.135997
H	-0.456023	2.295190	-1.223021
C	-0.960429	0.275140	-0.970669
H	-0.496506	-0.546674	-0.415694
H	-1.058694	-0.072381	-2.002892
C	-4.650258	-0.279461	0.032543
H	-4.579338	0.041611	1.078973
H	-5.081141	0.565577	-0.521328

$NpentylS^{-}$
E=-1142.7403848
G=-1142.497564

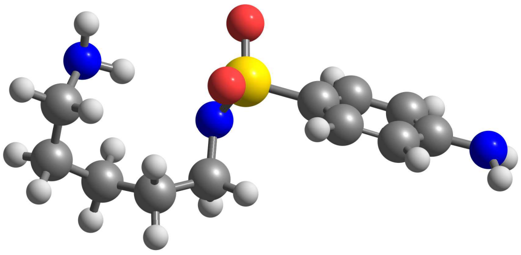

S	0.061671	-0.886374	-0.007554
O	0.067978	-2.265290	-0.525197
O	0.517040	-0.792810	1.395709
N	-5.674435	0.744502	0.098050
C	-1.656929	-0.402584	0.063187
C	-2.437407	-0.530927	-1.081538
C	-2.219725	0.142015	1.207324
C	-4.336601	0.402976	0.076806
C	-3.759670	-0.140416	-1.077033
C	-3.544361	0.542418	1.216926
H	-2.005222	-0.942939	-1.986979
H	-1.618599	0.253242	2.101119
H	-4.366318	-0.248802	-1.969959
H	-3.979342	0.969143	2.114499

	H	-6.083158	0.942782	-0.803569
	H	-5.936572	1.417419	0.803539
	N	0.802863	0.022778	-1.024103
	C	2.212530	1.735982	0.122874
	H	2.230319	1.132384	1.034506
	H	2.196716	2.782144	0.449518
	C	3.457027	1.470573	-0.719526
	H	3.211968	0.737135	-1.494157
	H	3.738599	2.381841	-1.256938
	C	4.411480	-0.355137	0.780178
	H	5.351733	-0.700393	1.217616
	H	3.726116	-0.196028	1.624659
	N	3.926200	-1.387672	-0.137862
	H	2.985407	-1.135158	-0.440194
	H	3.822756	-2.255892	0.376056
	C	0.925001	1.425009	-0.624015
	H	0.067314	1.765197	-0.022891
	H	0.898929	2.028689	-1.540050
	C	4.656567	0.961846	0.067690
	H	5.505776	0.842193	-0.615054
	H	4.956829	1.706044	0.815209
NhexylSH$_2^+$<br>E=-1183.4151303<br>G= -1183.102050<br>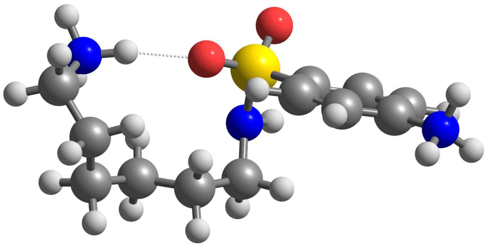	S	0.155184	-1.415049	-0.375088
	O	0.473878	-2.744273	-0.842043
	O	-0.825008	-0.638347	-1.101653
	N	5.179597	1.766301	-0.151132
	C	1.643265	-0.453466	-0.328364
	C	2.777773	-1.035572	0.217980
	C	1.642126	0.849369	-0.794098
	C	3.938017	0.994952	-0.197026
	C	3.944719	-0.299649	0.284158
	C	2.812842	1.585267	-0.731020
	H	2.755306	-2.052728	0.590260
	H	0.742639	1.288049	-1.205353
	H	4.847399	-0.724516	0.705490
	H	2.845936	2.606686	-1.089132
	H	5.785496	1.454129	0.612220
	H	4.994372	2.764367	-0.018806
	N	-0.347992	-1.598640	1.175723
	C	-1.276630	0.676337	1.698444
	H	-0.933937	1.129881	0.763766
	H	-1.179693	1.456706	2.460686
	C	-2.731279	0.256758	1.590671
	H	-2.804909	-0.638903	0.966689
	H	-3.081767	-0.048737	2.582679
	C	-4.139686	1.020801	-1.427363
	H	-5.216190	1.066760	-1.263220
	H	-3.926403	1.406576	-2.422612
	N	-3.777060	-0.431205	-1.450140
	H	-4.144686	-0.881328	-2.288686
	H	-4.172915	-0.919614	-0.645689

	H	-2.756141	-0.565499	-1.427144
	H	0.141682	-2.397944	1.570756
	H	5.709484	1.666805	-1.022923
	C	-0.343048	-0.442725	2.097171
	H	0.676197	-0.059991	2.221327
	H	-0.650499	-0.857584	3.059379
	C	-3.632307	1.370502	1.068619
	H	-4.682853	1.076179	1.175368
	H	-3.495850	2.242845	1.716364
	C	-3.384742	1.797400	-0.377365
	H	-3.698986	2.836243	-0.506498
	H	-2.315707	1.778314	-0.615954
NhexylSH$^+$<br>E=-1182.9801215<br>G=-1182.684133<br>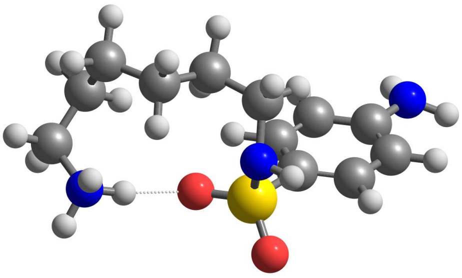	S	-0.207362	1.402156	-0.248078
	O	-0.490960	2.794857	-0.525882
	O	0.771220	0.724502	-1.079000
	N	-5.178410	-1.757379	-0.236927
	C	-1.683354	0.469061	-0.307334
	C	-2.824681	0.988530	0.300059
	C	-1.708823	-0.792862	-0.889082
	C	-4.034593	-1.014470	-0.284198
	C	-3.987689	0.256951	0.309063
	C	-2.875266	-1.524796	-0.883047
	H	-2.800499	1.968676	0.763446
	H	-0.817254	-1.196570	-1.352650
	H	-4.882063	0.656159	0.773447
	H	-2.906888	-2.505626	-1.343666
	H	-5.257600	-2.526040	-0.884856
	N	0.342648	1.390632	1.311175
	C	1.235238	-0.938176	1.556873
	H	0.926662	-1.236077	0.550720
	H	1.096669	-1.823896	2.185799
	C	2.697724	-0.530965	1.576719
	H	2.801836	0.466632	1.140802
	H	3.015460	-0.424515	2.619779
	C	4.159694	-0.677243	-1.477375
	H	5.230962	-0.692367	-1.276597
	H	4.005284	-0.883485	-2.535012
	N	3.704742	0.731406	-1.253030
	H	4.056621	1.345142	-1.987697
	H	4.050422	1.091354	-0.362593
	H	2.674155	0.796107	-1.237542
	H	-0.138575	2.138286	1.804952
	H	-6.039423	-1.267977	-0.047247
	C	0.300818	0.129660	2.077718
	H	-0.724349	-0.255717	2.126601
	H	0.588368	0.410363	3.093510
	C	3.610852	-1.535803	0.883675
	H	4.657979	-1.279462	1.082551
	H	3.445465	-2.514684	1.346415
	C	3.420159	-1.678141	-0.624307

	H	3.791033	-2.656373	-0.941195
	H	2.358125	-1.662390	-0.891760
NhexylS-open/amine<br>E=-1182.4992619<br>G= -1182.217267<br>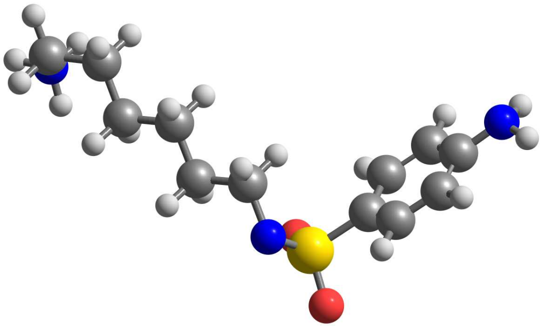	S	1.381850	1.691869	0.316621
	O	2.213052	2.907516	0.328305
	O	0.659181	1.464620	1.585830
	N	5.188935	-2.876759	-0.158750
	C	2.532614	0.326134	0.228685
	C	3.477265	0.311436	-0.792779
	C	2.488307	-0.732464	1.122955
	C	4.331966	-1.803977	-0.008090
	C	4.369493	-0.733228	-0.908881
	C	3.376693	-1.787672	1.008644
	H	3.519431	1.131327	-1.501659
	H	1.758161	-0.729979	1.922526
	H	5.110821	-0.735571	-1.701138
	H	3.339481	-2.612646	1.712344
	H	5.346558	-3.421685	0.676446
	N	0.505775	1.700148	-0.964513
	C	-1.813702	0.846891	-0.605287
	H	-2.201168	1.720830	-1.144292
	H	-1.792770	1.121504	0.455707
	C	-2.743329	-0.331879	-0.812934
	H	-2.756360	-0.607584	-1.875766
	H	-2.351809	-1.207350	-0.278068
	C	-6.522156	-0.990790	-0.224689
	H	-6.908692	-0.108476	-0.734574
	H	-7.153904	-1.841345	-0.472529
	N	-6.697120	-0.748699	1.241634
	H	-7.682699	-0.642100	1.482815
	H	-6.209282	0.096616	1.542342
	H	6.041239	-2.678180	-0.662294
	C	-0.401403	0.562092	-1.078297
	H	-0.030304	-0.332343	-0.550954
	H	-0.447481	0.272434	-2.135616
	C	-4.161510	-0.058058	-0.351455
	H	-4.137080	0.222849	0.709306
	H	-4.563556	0.809365	-0.890493
	C	-5.078114	-1.248445	-0.566251
	H	-5.047477	-1.544523	-1.619473
	H	-4.718195	-2.111749	0.006721
	H	-6.322247	-1.531446	1.781298
NhexylS-closed/amine<br>E=-1182.5035981<br>G=-1182.217900<br>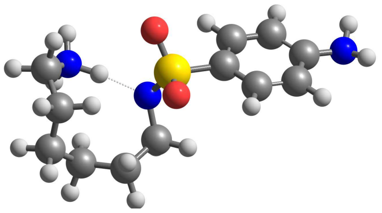	S	-0.084981	-0.432673	0.627325
	O	0.136818	-1.879192	0.763064
	O	0.150468	0.333511	1.862567
	N	-5.850970	0.304389	-0.654326
	C	-1.821139	-0.220580	0.296357
	C	-2.415954	-0.990540	-0.698260
	C	-2.582096	0.718735	0.976107
	C	-4.528139	0.110812	-0.315870
	C	-3.751546	-0.833611	-0.998513

C	-3.921675	0.885352	0.674374
H	-1.828818	-1.725453	-1.238007
H	-2.126810	1.320413	1.752697
H	-4.215427	-1.442227	-1.767328
H	-4.514723	1.619355	1.209502
H	-6.424036	0.729204	0.059934
N	0.724981	0.047762	-0.617382
C	2.080249	2.128151	-0.299466
H	2.133472	2.002733	0.786825
H	2.010673	3.206528	-0.474979
C	3.350342	1.576049	-0.960921
H	3.071469	0.899727	-1.775526
H	3.888276	2.392046	-1.451840
C	3.785211	-1.615190	0.095410
H	4.781962	-1.914493	-0.230774
H	3.401602	-2.387037	0.761789
N	2.904607	-1.616431	-1.110535
H	2.643084	-2.567741	-1.363162
H	3.387750	-1.209547	-1.910474
H	2.025122	-1.039639	-0.935066
H	-6.301172	-0.476839	-1.108418
C	0.795494	1.495447	-0.812917
H	-0.065088	2.012077	-0.364338
H	0.725877	1.688146	-1.890597
C	4.333050	0.903854	-0.002869
H	5.225609	0.592039	-0.559440
H	4.672321	1.666793	0.706220
C	3.814710	-0.281022	0.804454
H	4.467363	-0.428470	1.669559
H	2.821544	-0.064169	1.209559

NhexylS-closed/TS
E=-1182.5012885
G= -1182.219744

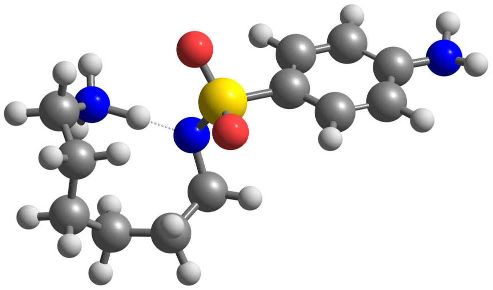

S	-0.080914	-0.410765	0.719599
O	0.189999	-1.832732	0.945734
O	0.105116	0.450343	1.892707
N	-5.795217	0.086493	-0.824501
C	-1.797935	-0.261813	0.302227
C	-2.329064	-1.103990	-0.669828
C	-2.609787	0.689028	0.902679
C	-4.484250	-0.048032	-0.427104
C	-3.654990	-1.004051	-1.027886
C	-3.939878	0.797049	0.542594
H	-1.700346	-1.848264	-1.145890
H	-2.201334	1.345371	1.660769
H	-4.070909	-1.666206	-1.779537
H	-4.574792	1.539655	1.013695
H	-6.409671	0.538974	-0.163941
N	0.759239	0.014442	-0.541622
C	2.030057	2.171925	-0.371043
H	2.075632	2.122316	0.721867
H	1.900387	3.229804	-0.619763
C	3.339321	1.655123	-0.982871

H	3.110867	0.968232	-1.804481
H	3.868201	2.486413	-1.457627
C	3.743888	-1.496620	0.111469
H	4.735264	-1.788719	-0.242828
H	3.402252	-2.267759	0.802621
N	2.806681	-1.502306	-1.040599
H	2.516917	-2.452715	-1.254564
H	3.261109	-1.132865	-1.872264
H	1.845051	-0.827771	-0.813846
H	-6.206227	-0.724686	-1.262572
C	0.788440	1.442211	-0.858838
H	-0.106819	1.946391	-0.472118
H	0.735625	1.540019	-1.949556
C	4.310804	1.017879	0.009302
H	5.220725	0.715754	-0.524092
H	4.620088	1.799725	0.712076
C	3.804785	-0.164196	0.826542
H	4.481827	-0.313004	1.672977
H	2.826593	0.063191	1.261544

NhexylS-closed/sulfonamide
E=-1182.5117427
G=-1182.227924

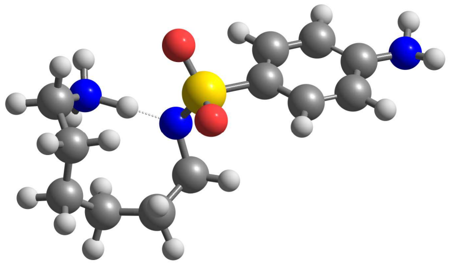

S	0.146892	-0.455373	-0.687616
O	-0.135655	-1.874364	-0.819165
O	-0.144611	0.388781	-1.834257
N	5.846850	0.277641	0.665883
C	1.840941	-0.222899	-0.298207
C	2.434316	-1.042992	0.657945
C	2.588631	0.755447	-0.939704
C	4.534446	0.095595	0.324733
C	3.765111	-0.888140	0.964598
C	3.923118	0.914390	-0.631248
H	1.851798	-1.807406	1.159225
H	2.125318	1.390049	-1.684323
H	4.234864	-1.527383	1.703674
H	4.512475	1.675607	-1.130061
H	6.423839	0.776166	0.005489
N	-0.647146	0.014860	0.656159
C	-2.030318	2.094504	0.317892
H	-2.033915	1.951268	-0.766709
H	-1.936410	3.172259	0.478151
C	-3.344571	1.590755	0.932204
H	-3.124469	0.895813	1.747993
H	-3.861604	2.428991	1.407653
C	-3.932086	-1.587060	-0.051195
H	-4.990074	-1.784130	0.172210
H	-3.596240	-2.397407	-0.703841
N	-3.104463	-1.628375	1.162256
H	-3.050787	-2.586300	1.490225
H	-3.569324	-1.111628	1.901889
H	-1.482716	-0.576228	0.818238
H	6.307832	-0.492336	1.126471
C	-0.787433	1.453407	0.905997

	H	0.113938	1.948595	0.532767
	H	-0.782674	1.592472	1.990958
	C	-4.311282	0.960523	-0.067380
	H	-5.250320	0.717298	0.446039
	H	-4.564764	1.728957	-0.806860
	C	-3.826606	-0.279884	-0.811259
	H	-4.430512	-0.395817	-1.717215
	H	-2.796683	-0.138966	-1.159091
NhexylS-open/sulfonamide<br>E=-1182.5138003<br>G=-1182.235058<br>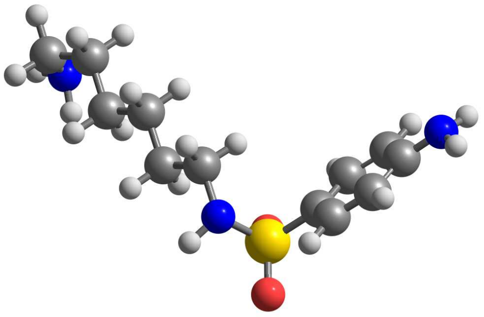	S	-1.334122	-1.597993	0.383781
	O	-1.973043	-2.897453	0.292470
	O	-0.542776	-1.324635	1.569417
	N	-5.357629	2.677609	-0.150335
	C	-2.521043	-0.322286	0.235955
	C	-3.517566	-0.429704	-0.731370
	C	-2.480827	0.778794	1.082064
	C	-4.440329	1.676175	-0.001941
	C	-4.466875	0.556689	-0.848440
	C	-3.431383	1.768919	0.964585
	H	-3.550414	-1.289946	-1.389972
	H	-1.705804	0.857085	1.833780
	H	-5.249148	0.479287	-1.595205
	H	-3.407915	2.630572	1.622305
	H	-5.476299	3.310174	0.626137
	N	-0.456784	-1.466134	-1.004221
	C	1.890562	-0.701483	-0.603463
	H	2.256743	-1.576789	-1.154890
	H	1.848831	-0.990341	0.451999
	C	2.852319	0.455620	-0.785261
	H	2.879802	0.746247	-1.843541
	H	2.476073	1.331082	-0.239847
	C	6.643022	0.976675	-0.117192
	H	6.995763	0.157857	-0.762128
	H	7.279053	1.838170	-0.334508
	N	6.780820	0.665824	1.306921
	H	7.758287	0.491982	1.513038
	H	6.302794	-0.207328	1.504312
	H	-0.059831	-2.374499	-1.234687
	H	-6.205588	2.453549	-0.648110
	C	0.503115	-0.359176	-1.096363
	H	0.094483	0.493519	-0.544460
	H	0.545578	-0.051010	-2.144684
	C	4.257505	0.135645	-0.314127
	H	4.215723	-0.169297	0.738966
	H	4.640079	-0.731760	-0.869420
	C	5.213608	1.301650	-0.486441
	H	5.190574	1.627665	-1.533132
	H	4.866216	2.152172	0.113848
NhexylS^{-}<br>E=-1182.0295028<br>G=-1181.760416	S	0.215327	-1.334930	-0.064013
	O	0.511631	-2.748705	-0.358335
	O	-0.651084	-0.728403	-1.100102

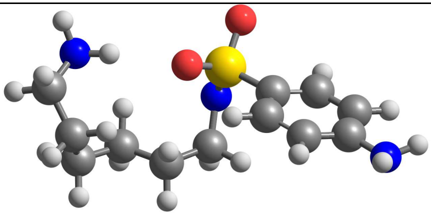

N	5.381418	1.624054	-0.345102
C	1.761307	-0.457055	-0.217860
C	2.882786	-0.952603	0.439011
C	1.857886	0.736861	-0.916116
C	4.192229	0.922033	-0.330238
C	4.084637	-0.278762	0.380528
C	3.059324	1.420585	-0.975265
H	2.816000	-1.880640	0.996315
H	0.988193	1.133395	-1.425801
H	4.959883	-0.674994	0.884308
H	3.131726	2.351190	-1.528080
H	5.493537	2.264034	-1.117933
N	-0.275608	-1.230268	1.403706
C	-1.406720	1.055242	1.421617
H	-1.150213	1.306444	0.387311
H	-1.347693	1.994434	1.987639
C	-2.822133	0.514003	1.497410
H	-2.854970	-0.480173	1.046863
H	-3.087545	0.382851	2.554286
C	-4.469743	0.431623	-1.471779
H	-5.533412	0.446545	-1.206549
H	-4.415987	0.691857	-2.538324
N	-3.946097	-0.909694	-1.204611
H	-2.931118	-0.892897	-1.282935
H	-4.278520	-1.544337	-1.921404
H	6.208735	1.071828	-0.171874
C	-0.360401	0.105370	1.985701
H	0.618016	0.612218	1.953806
H	-0.577461	-0.043073	3.049797
C	-3.855375	1.413386	0.829927
H	-4.864578	1.069296	1.088875
H	-3.760855	2.418626	1.259200
C	-3.753999	1.514561	-0.690742
H	-4.178555	2.470331	-1.014416
H	-2.701579	1.538705	-1.000499

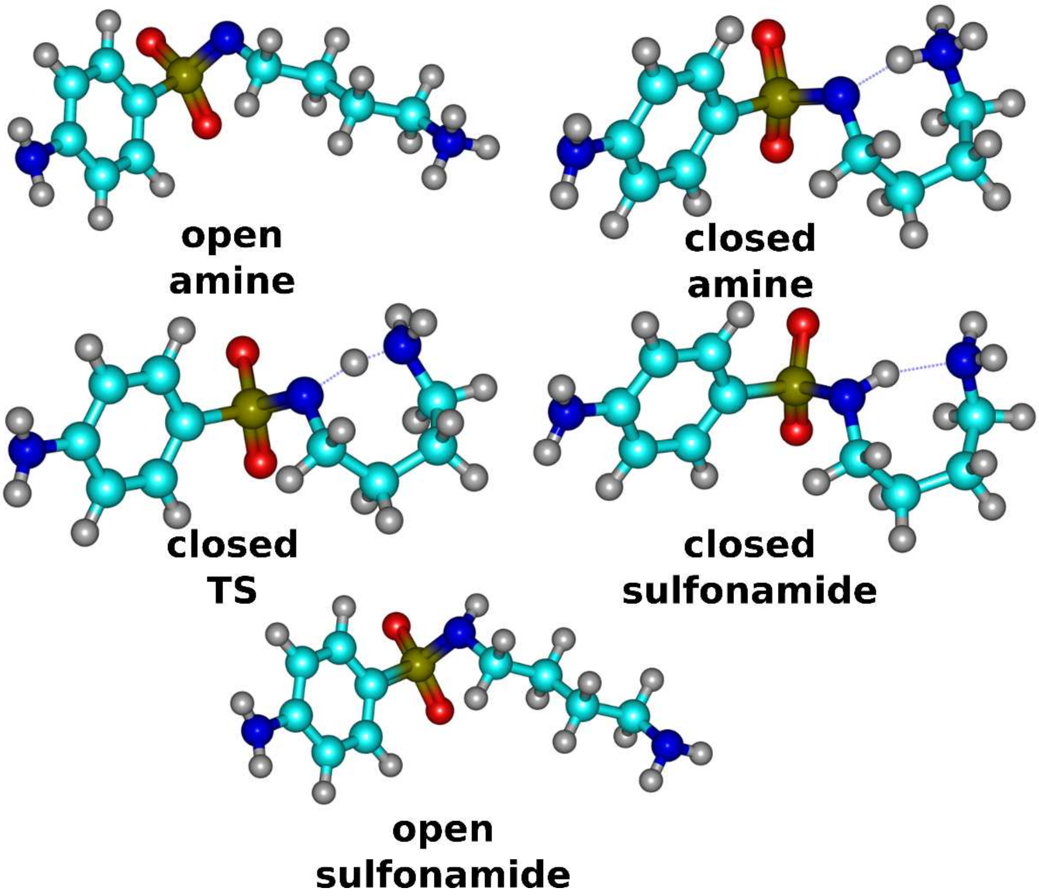

Figure S1. The equilibrium structures of various forms of zwitterions sulfonamides. Colored circles, light blue, blue, red, yellow, and grey refer to C, N, O, S, and H atoms, respectively.

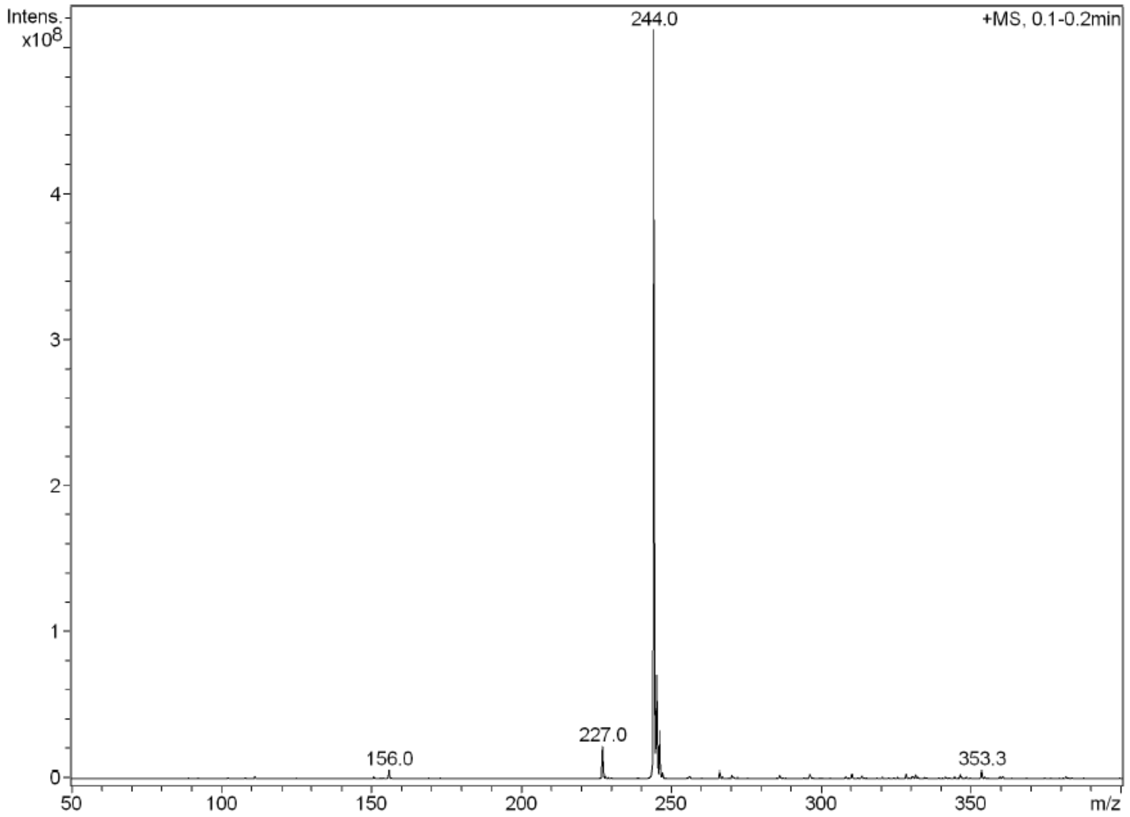

Figure S2. Mass spectrum for NbutylS.

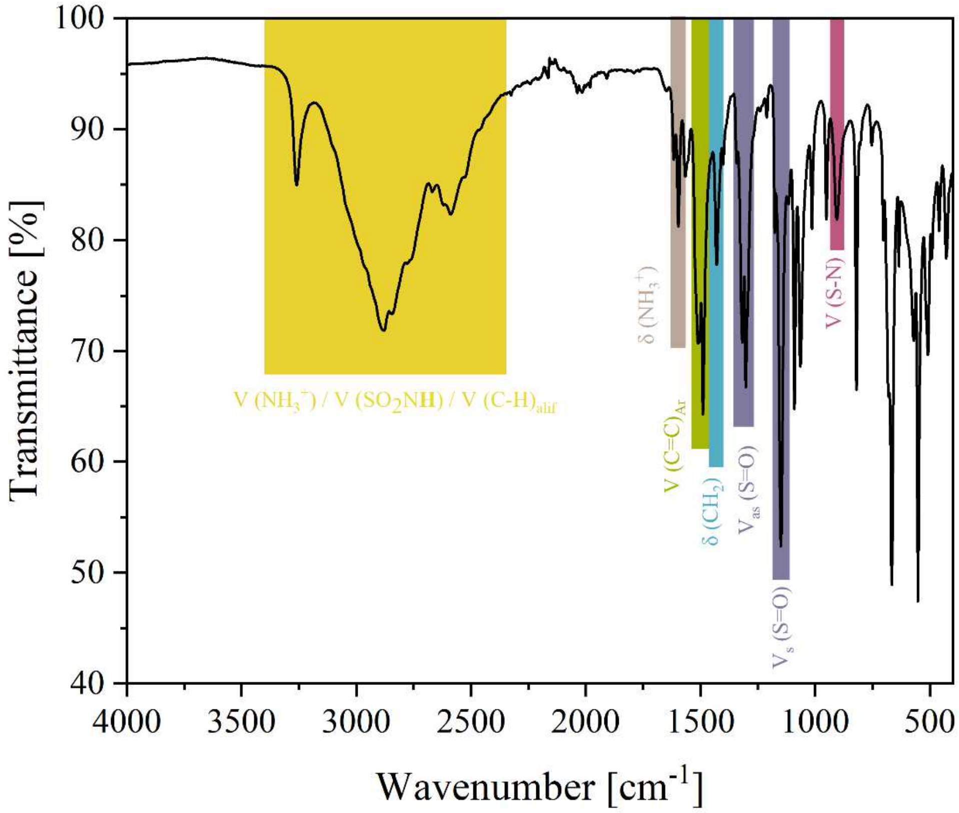

Figure S3. The FT-IR spectra for NbutylS.

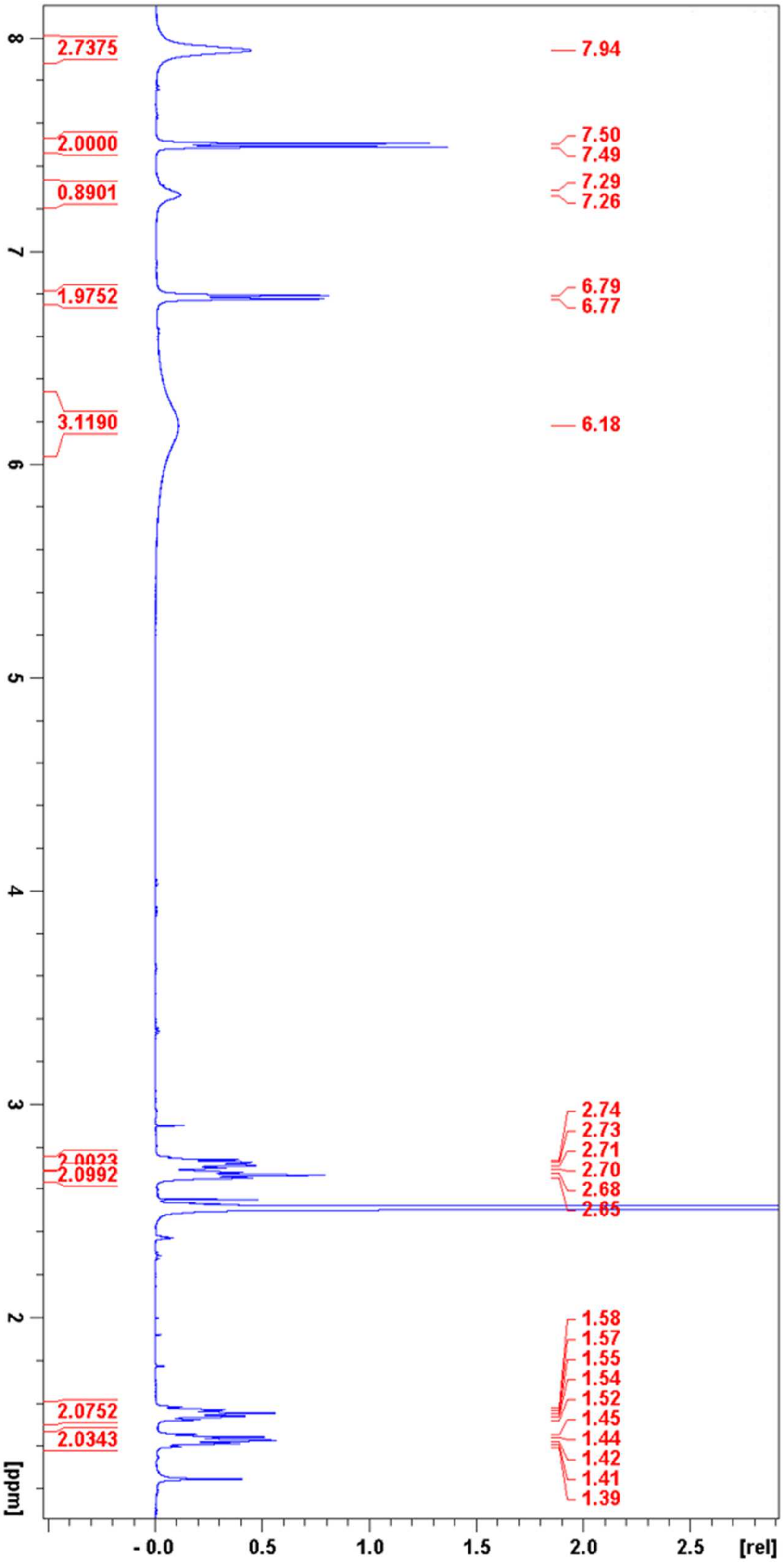

Figure S4. ^{1}H NMR spectra for NbutylS.

ELEMENTAL ANALYSIS DATA

Anal. Calcd. (%) for $C_{10}H_{19}N_3O_2SCl_2$ (316.29): C, 37.98; H, 6.06; N, 13.29; S, 10.14;

Found (%): C, 37.90; H, 6.125; N, 12.85; S, 9.85;

ESI-MS *m/z*: 244.0 [M + H];

FT-IR ATR (cm^{-1}) selected bands: 3345-2326 (m) ν(NH of $-NH^{3+}$)/ν(NH of SO_2NH)/ $\nu(C\text{-}H)_{alif}$; 1595 (w) δ(NH^{3+}); 1515 (m), 1485 (m) $\nu(C{=}C)_{Ar}$; 1426 (w) δ(CH of CH_2); 1299 (m) $\nu_{as}(S{=}O)$; 1143 (s) $\nu_s(S{=}O)$; 902 (w) ν(N-S);

^{1}H NMR (DMSO-*d*$_6$) *δ* 1.42 (qu, 2H, *J* = 2.93 Hz); 1.55 (qu, 2H, *J* = 7.61 Hz); 2.66 (t, 2H, *J* = 6.47 Hz); 2.70 (qu, 2H, *J* = 6.93 Hz); 6.18 (s, 3H, -N**H**); 6.78 (d, 2H, *J* = 8.62 Hz); 7.27 (s, 1H, $-SO_2N$**H**); 7.49 (d, 2H, *J* = 7.90 Hz); 7.94 (s, 3H, -N**H**).

The registered FT-IR spectrum (**Figure S3**) for the compound NbutylS contains characteristic vibration bands of the sulfonamide group $-SO_2NH-$ and the primary ammonium group $-NH^{3+}$. The observed wide band occurring in the range of 3345-2326 cm^{-1} was assigned by N_{single} bond H stretching vibrations of the primary ammonium group ($–NH_3^+$). There are also characteristic bands corresponding to asymmetric and symmetrical vibrations stretching the S=O bonds, occurring in the range of 1346-1114 cm^{-1}. The band of stretching vibrations of C=C aromatic rings is observed in the range of 1531-1468 cm^{-1}, while the stretching vibrations characteristic of the S-N bond are in the range of 929-843 cm^{-1}.

The presented ^{1}H NMR spectrum for NbutylS (**Figure S4**) indicates the presence of the characteristic sulfonamide moiety $-SO_2NH-$ for this group of compounds, the signal of which is observed at 7.27 ppm. Aliphatic atoms derived from the alkylamino chain are in the range of 1.39-1.58 and 2.65-2.74 ppm. Protons from ammonium groups ($-NH^{3+}$) appear as singlet peaks at 6.18 and 7.94 ppm. Aromatic protons are observed at 6.78 and 7.49 ppm.
